# Taming Waste Heterogeneity for Plastics Circularity with Optimized Sample Preparation Protocols for Quality Assessment

**DOI:** 10.3390/polym18030409

**Published:** 2026-02-04

**Authors:** Christos Panagiotopoulos, Christina Podara, Eleni Gkartzou, Melpo Karamitrou, Tatjana Kosanovic-Milickovic, Mara Silber, Lars Meyer, Bernhard von Vacano, Ana Rita Carvalho Neiva, Jan-Hendrik Knoop, Asunción Martínez-García, Ana Ibáñez-García, Silvia Pavlidou, Leila Poudeh, Costas A. Charitidis, Stamatina N. Vouyiouka

**Affiliations:** 1 Laboratory of Polymer Technology, School of Chemical Engineering, Zographou Campus, National Technical University of Athens, 157 72 Athens, Greece; chpanagiotopoulos@mail.ntua.gr; 2 Research Lab of Advanced, Composites, Nanomaterials and Nanotechnology, School of Chemical Engineering, Zographou Campus, National Technical University of Athens, 157 72 Athens, Greece; cpodara@chemeng.ntua.gr (C.P.); egartzou@chemeng.ntua.gr (E.G.); mkaramitru@chemeng.ntua.gr (M.K.); tkosanovic@chemeng.ntua.gr (T.K.-M.); 3 Group Research, BASF SE, 67056 Ludwigshafen am Rhein, Germany; mara.silber@basf.com (M.S.); lars.meyer@basf.com (L.M.); bernhard.von-vacano@basf.com (B.v.V.); 4 Coolrec Plastics BV, Van Hilststraat 7, 5145 RK Waalwijk, The Netherlands; Ana.Rita.Neiva@coolrec.com; 5 Fraunhofer Institute for Process Engineering and Packaging IVV, 85354 Freising, Germany; jan-hendrik.knoop@ivv.fraunhofer.de; 6 Innovative Materials and Manufacturing Area, AIJU, Technological Institute for Children’s Products and Leisure, 03440 Ibi, Alicante, Spain; sunymartinez@aiju.es (A.M.-G.); anaibanyez@aiju.es (A.I.-G.); 7 MIRTEC S.A., 76th km Athens-Lamia National Road, 320 09 Schimatari, Greece; s.pavlidou@mirtec.gr; 8 Central R&D, Polymer & Chemistry Department, Beko Corporate (Arçelik Global), 34950 Tuzla, Istanbul, Turkey; leila.poudeh@beko.com

**Keywords:** recycling, upcycling, polymer waste, homogeneity, sustainability, processability

## Abstract

From the perspective of the circular economy and minimization of environmental pollution, recycling plastics is key for transforming polymeric waste streams (PWSs) towards reusable and, if possible, upgraded, value-added products. The low homogeneity of PWSs, even when sorted, complicates sampling, analytical characterization, processability, and quality assurance of the whole circular process. Therefore, sampling, sample preparation, and analysis methodologies that yield results accurate and representative enough to describe the contents and the safety of the bulk while being cost-effective are crucial. In this context, an experimental “model waste” approach was conceptualized to reliably assess and optimize sampling and sample preparation strategies towards specific goals, i.e., identifying and precisely quantifying different polymer types and non-polymeric contaminants (such as brominated flame retardants, BFR) along with establishing a correlation of the sample preparation steps with low deviation values between replicates. The results indicated that cryogenic grinding better preserved additive content, minimizing its degradation, i.e., 461 ± 17 ppm determined via HPLC-MS when the nominal concentration was 500 ppm. On the other hand, melt-based homogenization significantly improved homogeneity and hence reproducibility/variability of analytical results (RSD), albeit at the risk of partial additive thermal degradation (up to 70% reduction in BFR content). The current experimental approach allows a clear understanding of plastic waste characteristics in view of demonstrating analytical limits of detection (LoD), reliable verification of compliance with certain concentrations of unwanted contaminants, and eventually robust evaluation of the applied recycling scheme efficiency.

## 1. Introduction

Polymeric materials—plastics—are indispensable in today’s world for their performance and efficiency, produced at high scale and low cost. However, their utility creates large volumes of polymer waste streams (PWSs), environmental pollution, and CO_2_ emissions during their fossil-based life cycle [1]. Next to reduction and reuse strategies, recycling is decisive to create a circular plastics economy and valorize the waste. While recycling approaches have been developed and scaled [2], they so far fall short: less than 10% of plastics are currently being recycled at least once [3], while in some geographies as in the EU, higher rates of 19% are achieved across all plastics applications [4]. A big limitation today, next to the lack of collection and sorting infrastructure, is deficiencies in consistent recyclate quality: being made from heterogeneous waste streams [5,6,7], as well as widely varying contents of minor components or contaminants, high-quality recyclates remain challenging.

High-quality recycling requires, above all, a thorough understanding of the inhomogeneous PWS input. The inhomogeneity depends (i) on the intrinsic material complexity of the initial product, i.e., waste from electrical and electronic equipment (WEEE), vs. toys or vs. textiles, (ii) the geographic origin, (iii) the efficiency of collection and sorting/separation steps, (iv) the statistical distribution of waste objects in the respective batch, as well as (iv) variations within the same waste batch. A waste sample *s*_i_, would be thus, a distribution of *n* objects or particles with *composition*_i,*n*_ (diverse polymer types with different degradation extent, non-polymeric contaminants, including pigments, organic residues, metals, wood, rubber, legacy additives, etc.) and a specific *shape*_i,*n*_ (e.g., particle size and morphology), forming the distribution as follows:

$$s_{i}=\left[\begin{array}{cc} shape_{i,1} & composition_{i,1}\\ shape_{i,2} & composition_{i,2}\\ \ldots & \ldots \\ shape_{i,n} & composition_{i,n} \end{array}\right]$$

**Figure 1:**
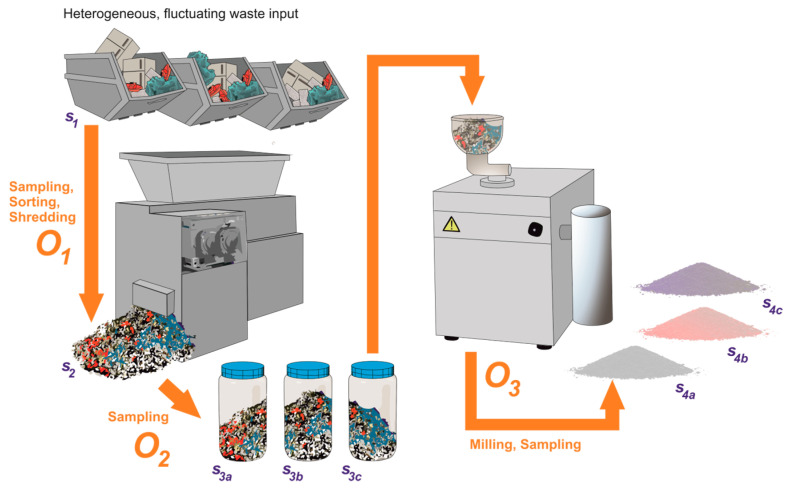
Simplified, exemplary sampling and sample preparation workflow from heterogeneous, waste input for later chemical analysis at the milligram scale. An initial sample *s*_1_ is processed and further downsampled by sampling, sorting, and shredding (*O*_1_), selection of a sub-sample (*O*_2_), further homogenization by milling, and more sampling (*O*_3_). Due to the many unknown and complicated dependencies in the whole chain of *O*_1_, *O*_2_, and *O*_3_.

The inhomogeneous PWS undergoes separation/sorting to the best possible extent, but the derived sorted plastic waste—and the respective plastic recyclate—will always have an inherently inhomogeneous nature, exacerbated by large quantities produced in sorting industrial plants and by both waste and recyclate composition likely differing between different sources of input materials or over time. Regarding sorted waste characterization, it will never be possible to analyze the whole waste input, and different analytical tools can only accept very limited sample qualities. Meaningful and representative results rely on sampling strategies, i.e., careful selection of samples and meticulous sample preparation techniques (Figure 1). These sample preparation steps (operations *O*) each create new samples *s*_i + 1_ from an initial sample *s*_i_:

$$s_{i+1}=O_{l}(s_{i})$$

Subsequent steps would further change the distribution and select a subset, such as the following:

$$s_{i+2}=O_{2}(s_{i+1})=O_{2}(O_{l}(s_{i}))$$

If the nature of these operations *O*_1_, *O*_2_, … is exactly known and mathematically described, the sampling can be completely guided by mathematical statistical considerations. There are standards and protocols that provide guidelines for sampling in industrial scale based on the material properties and analytical objectives (DIN CEN/TR 15310 [8,9,10,11,12], DIN CEN/TS 16010 [13] and 16011 [14], EN 14899 [15], ASTM D4687 [16] and D7204 [17]). However, they generally assume a simple, known behavior of sampling operations *O* and/or homogeneous material on the respective object level; hence, a single measurement is sufficient for characterization. More importantly, they do not correlate the sampling step with specific analytical tools, and they do not recommend how to obtain comparatively very small, but representative amounts for laboratory analysis. On the other hand, analyzing large sample sizes and/or a great number of replicates can be effective for laboratory analysis, but it is not always feasible; time and cost, sample morphology and amount needed for specific analytical techniques, limit of detection (LoD), etc., are limiting factors. Additionally, sample preparation steps (operations *O*) may have complex outcomes in practice. To name a few: rubbery particles might not be effectively reduced in size in shredding or milling, while hard plastic particles would. Volatile compounds could be lost or redistributed differently if different materials experience different heat stress, and so on.

As a result of the aforementioned limitations in the sampling and sample preparation steps, ensuring that the recyclates comply with the requirements imposed by stakeholders, legal authorities, manufacturers, and consumers—such as Toys Safety Directive (TSD) [18], Restriction of Hazardous Substances in Electrical and Electronic Equipment (RoHS) [19], OEKO-TEX for textiles [20], and food contact demands [21]—is quite challenging: it highly depends on establishing sampling and sample preparation schemes that would consider both the inhomogeneity of recyclates and practical limitations from a laboratory standpoint. To address these challenges, the current study proposes an experimental approach to allow a clear understanding of plastic waste characteristics through an optimal combination of *n* operations *O*, which leads to a final representative sample *s*_final_ = *O*_n_(…*O*_2_(*O*_1_(*s*_initial_))). This approach is based on an input waste of known composition and contaminants (a “model waste”), and the validity of the whole sampling and sample preparation chain, together with the selection of analytical methods, is investigated, targeting reliable verification of material compliance with specific regulatory requirements imposed by the EU, e.g., with required concentration limits.

More specifically, a model mixture (“model waste”) of polymers (ABS, HIPS, PP, and HDPE) was prepared targeting a specific and well-defined waste stream, namely plastics from end-of-life refrigerators. The predefined composition was chosen based on the average recycled ABS obtained from the mechanical sorting of WEEE: the different grades of recycled ABS released into the market are expected to have a purity level > 90%, with polymeric contamination from HIPS and PP/PE [22]. Even more, ABS-rich waste streams need high operating temperatures (190–240 °C) [23,24,25] for melt-based processes (e.g., extrusion, injection) and thus any thermal degradation of the additives (flame retardants, lubricants, etc.) and/or other polymer types, and its subsequent effects upon the analytical tools can be easier to observe and quantify.

The second type of analyte considered herein was small molecules, like additives, degradation products, or non-intentionally added substances. This very broad range of molecules is not distributed equally over all flakes, but more likely distinct molecules are present in individual flakes, depending on the origin of each material. To mimic this, a brominated flame retardant (FR), tetrabromobisphenol A (TBBPA), commonly used in electronics, was incorporated in the polymeric mixture at 500 and 50 ppm, to reflect both higher contamination levels in older devices and lower concentrations aligned with tightening regulatory limits. These concentrations help simulate the challenges of detecting toxic substances in a complex, heterogeneous matrix, where inconsistent particle size, distribution, and the variability of recycling processes further hinder accurate analysis. As European regulations continue to lower the allowable limits for hazardous substances in WEEE [26], TBBPA presence at low and continuously decreasing concentration thresholds renders it well-suited for assessing analytical limits of detection, inter-replicate variability, and the risk of underestimation associated with inappropriate homogenization, degradation/volatilization during sample preparation, etc.

The effect of different blending/homogenization methods (cryogenic grinding/milling, extrusion, injection molding, and their combination) upon the ability to detect (non)polymeric contaminants and to obtain consistent and accurate results was herein investigated. Analyzing the model waste using established laboratory methods aspires to gain valuable insights into the potentials and limitations of each analytical method towards sustainable and safe recycling schemes.

## 2. Materials and Methods

### 2.1. Polymeric Materials and Model Waste Composition

In order to demonstrate the challenges of sampling and sample preparation in plastics recycling, a real PP waste coming from refrigerators (PP-RE, Figure S1) was used as a reference and was thoroughly characterized. The PP-RE was sorted at Coolrec Plastics Plant (Waalwijk, The Netherlands), yielding a PP-rich material (~98% purity) in the form of flakes (2–4 mm, thickness 1–2 mm). Its composition in different polymer types was determined via NIR spectroscopy at the Coolrec Plastics Plant. For the model waste study, an ABS-rich polymeric blend was considered using commercially available virgin polymers with the following mass fractions (% wt.). Acrylonitrile butadiene styrene, ABS (90.95% wt., ABS Kumho 750SW, Kumho Petrochemical Co. Ltd., Seoul, Republic of Korea), high-impact polystyrene, HIPS (6% wt., HIPS styrolution PS 495N, Trinseo, Wayne, PA, USA), polypropylene, PP (2% wt., PP Borealis HE 125MO, Borealis AG, Vienna, Austria), and high-density polyethylene, HDPE (1% wt., HDPE Petkim Petilen YY S0464, Petkim Petrokimya Holding A.Ş., Izmir, Turkey). Also, a masterbatch of ABS with 10% wt. of the chosen brominated flame retardant, TBBPA (3,3′,5,5′-tetrabromobisphenol A, ICL Group Ltd., Tel Aviv, Israel), named as 10% wt. BFR-ABS was prepared and then added to the polymer blend in a sufficient amount to achieve a final concentration of 500 or 50 ppm TBBPA. The 10% wt. BFR-ABS was prepared using PRISM TSE 24 HC twin-screw co-rotating extruder (Thermo Scientific™, Bremen, Germany) with a screw diameter of 24 mm and L/D ratio 28:1 with a screw speed of 200 rpm. The temperature profile of the compounder was set to 190/200/225/235/235/225/210 °C.

### 2.2. Theoretical Approach to Evaluate Sample Preparation Techniques

A theoretical framework was created based on the ABS-rich waste stream (model waste) to assess the influence of particle size and sample mass on analytical accuracy and precision. Starting with 1 kg of model waste with the predefined composition (Table S1), uniform flake mass and particle size were assumed, yielding in total 20,000 flakes (average mass of 0.05 g per flake) or 400,000 (computationally ground/milled) particles mimicking a ring sieve mill of 750 µm, which were assigned to cells in excel based on each component share. Then, ten subsamples of 3 g each were theoretically analyzed by implementing a simple Monte-Carlo simulation that randomly picked a cell-polymer and calculated its share in percentage. The calculated percentage of the polymers and non-polymeric contaminant (TBBPA) for the case of flakes vs. particles was depicted in bar charts, and different sample sizes (0.1, 1, 5, and 10 g) were simulated and depicted in box plots in order to assess the influence of sample size upon the analytical accuracy.

### 2.3. Preparation of the Model Waste Samples

The aforementioned ABS-rich mixture containing TBBPA was prepared via different blending/homogenization procedures to prepare model waste samples (Table 1) that were afterwards analyzed: (i) cryogenic grinding in a centrifugal mill (ZM 300, Retsch, Haan, Germany) equipped with a 750 μm pore size sieve using a combination of dry ice and liquid N_2_ achieving an average particle size of 600 μm, (ii) extrusion (Collin ZK25 twin-screw co-rotating extruder, Lab & Pilot Solutions GmbH, Maitenbeth, Germany) at 130 rpm with a temperature profile of 175/195/198/200/200/203/201 °C or 175/197/203/206/206/208/206 °C (from feeding zone to die), and measuring the temperature of the melt (*T*_melt_) equal to 200 or 210 °C, respectively, cooled in a water bath, dried at 80 °C for 2 h and subsequent pelletizing in a Collin Teach-Line CSG 171T pelletizer (Lab & Pilot Solutions GmbH, Maitenbeth, Germany), and (iii) the combination of the aforementioned methods with each other or with injection molding (Arburg Allrounder 370C machine, GmbH & Co KG, Loßburg, Germany) operating at the following conditions: barrel temperature profile 235/240/245/245 °C, injection speed of 60 cm^3^/s, injection time 0.6 s, maximum injection and holding pressure of 850 and 400 bar, respectively, holding, cooling and cycle time of 2, 6 and 13 s, respectively, specimens type according to ISO 527-2 1B [27].

Prior to any processing, the virgin polymers were dried for 2 h at 80 °C in an air-circulating oven to remove any moisture.

### 2.4. Characterization

#### 2.4.1. Attenuated Total Reflectance (ATR) Fourier Transform Infrared (FTIR) Spectroscopy

FTIR measurements were performed in an Alpha II (Alpha II, Bruker, Leipzig, Germany) using the Attenuated Total Reflectance (ATR) sampling accessory equipped with a diamond crystal. All the samples were analyzed at room temperature in the absorbance mode. For the sorted PP-rich waste stream (PP-RE), random flakes with different colors were directly tested in the ATR mode. For the model waste samples, five films of 80–100 μm thickness (each film 200 mg) were prepared for each sample of a different sampling approach by compression molding in a heated-plates press, operating at 150–200 °C, and 4 random spectra were collected from each film, for a total of 20 individual spectra. For all samples, the average of these 20 spectra was depicted in the following figures. Films are the preferred sample morphology vs. shredded particles or powder because diamond crystal-surface contact issues are reduced (baseline shifts, lower band intensities, low signal-to-noise ratio). A thin film was placed on the aperture, and 32 scans were performed over the spectral range of 400–4000 cm^−1^ at 4 cm^−1^ resolution and averaged across the spectral range to improve the signal-to-noise ratio. Also, OPUS Software^®^ (version 8.5) delivers atmospheric compensation for CO_2_ (in the 2282–2399 cm^−1^ region) and H_2_O (in the 1300–1400 cm^−1^ and 3667–3996 cm^−1^ regions).

ATR-FTIR spectra comparison methodology relied on the use of comparative charts, such as Tables S2, S3, S5 and S6. The mid-IR wavenumber region was divided into two separate ones: MWIR (middle wavelength IR region, 3100 to 2200 cm^−1^) and LWIR (long wavenumber IR region, 1600 to 600 cm^−1^). All the important peaks were collected within 10 cm^−1^ wide columns, with a deviation of ±5 cm^−1^. A color coding was applied to reflect the relative intensity of the signals within the considered range; blue, red, orange, yellow, and pale yellow correspond to the “outstandingly intense”, “strong”, “moderate”, “weak”, and “very weak, hard-to-see” mentions, often found in the literature. In the case of convoluted peaks and shoulders, the assigned color corresponded to the particularity of the peak. This way of representation facilitated the identification of signals and specific patterns characteristic of chemical species or polymer types present in waste streams by columns that are empty elsewhere. Also, because an isolated signal can correspond to several species, one can observe the complementary peaks in order to identify a polymer type present in a real waste stream. Baseline correction was deliberately omitted to preserve subtle details (e.g., small peaks and shoulders), particularly in the LWIR, where the baseline tends to increase, due to the intrinsic feature of ATR to increasingly penetrate the material in this region and therefore exhibit stronger absorptions.

#### 2.4.2. High-Performance Liquid Chromatography Coupled to Mass Spectrometry (HPLC-MS)

For the quantitative analysis of tetrabromobisphenol A (TBBPA), a Q Exactive™ Plus Orbitrap mass spectrometer (Thermo Scientific™, Bremen, Germany) was used in combination with an Ultimate 3000 ultra-high-performance liquid chromatography (UHPLC) system (Thermo Scientific™, Bremen, Germany). The Ultimate UHPLC system was configured with an LPG-3400RS quaternary pump, an ISO-3100SD isocratic pump, a WPS-3000TRS autosampler, and a TCC-3000SD column compartment. The reverse-phase liquid chromatography (RP-LC) for TBBPA analysis was conducted utilizing a Waters XSelect^®^ CSH™ C18 column (3.5 μm, 3.0 × 20 mm IS™) as a pre-column and a YMC Triart C18 ExRS column (50 × 3 mm, S-3 μm) as the separation column.

For the chromatographic separation, the eluents utilized consisted of 0.1% w/v formic acid in Millipore water (eluent A), 0.1% formic acid in acetonitrile (eluent B), and tetrahydrofuran (eluent C) for the quaternary pump. A flow rate of 0.5 mL/min was maintained throughout the analysis. The gradient commenced with a composition of 90% A and 10% B. Over the course of 1 to 16 min, the proportion of eluent A was gradually decreased to 0%, concomitantly increasing the proportions of eluent B to 90% and eluent C to 10%. Following this, eluent B was reduced to 0% while eluent C was elevated to 100% over a period of 10 min, and this condition was sustained for an additional 9 min. Subsequently, the gradient was reverted to the initial conditions of 90% A and 10% B within 0.5 min and maintained for the final 4.5 min.

The column oven was maintained at a temperature of 40 °C, and an injection volume of 5 μL was employed for the analysis. Tetrabromobisphenol A (TBBPA) was detected at a wavelength of 290 nm. Ionization of TBBPA was performed in negative ionization mode using atmospheric pressure chemical ionization (APCI). The MS parameters included a resolution of R = 35,000 at *m*/*z* = 200, a scan range of *m*/*z* = 450–650, an automatic gain control (AGC) target set at 1∙10^6^ ions, and a maximum injection time of 128 ms in Full MS mode. For data analysis, the Quant Browser application from Xcalibur 4.6 (Thermo Scientific) was utilized. TBBPA detection in MS was achieved by extracted ion chromatograms, which incorporated all relevant bromine isotopes at *m*/*z* = 538.7498, 540.7478, 542.7457, 544.7437, and 546.7416, with a mass deviation threshold of 10 milli mass units (mmu).

For sample preparation, approximately 1 g of each sample was precisely weighed to the nearest 0.01 mg and subsequently transferred into a 10 mL glass vial fitted with a screw cap. Following this, 8 mL of methanol was added to each sample. The samples were then mixed for a duration of 17.5 h using a roll mixer set to a rotational speed of 80 rpm. Afterwards, each sample was subjected to centrifugation for 10 min at room temperature, generating a centrifugal force of 2401× *g*. Subsequently, 1 mL of the resulting supernatant was carefully transferred into a 1.5 mL glass vial for injection into the HPLC-MS system.

#### 2.4.3. X-Ray Fluorescence Spectrometry (XRF)

XRF analyses were performed in order to characterize the samples for their elemental composition. Both the real sorted PP-rich waste stream (ΡΡ-RE) and the model waste were cryogenically ground for XRF analyses. The ground material was melted in the form of pellets (3–5 g each) to result in a homogeneous sample with minimized boundary surfaces and grain size effects. Five replicates were analyzed per sample. For XRF analysis, the SPECTRO XEPOS X-ray fluorescence spectrometer (XEP05, SPECTRO Analytical Instruments, Kleve, Germany) was used. XRF consists of a palladium end-window X-ray tube and a built-in lithium-drifted silicon semiconductor detector cooled with liquid nitrogen. The samples were irradiated several times in succession with the respective monochromatic X-ray radiation of a target, and a spectrum was generated from the detected radiation. For the evaluation, the spectra obtained were first subjected to background elimination. Subsequently, the spectra were deconvolved by dividing them into individual energy ranges. The position, half-width, and area of the detected peaks were determined, and the pulse number of a corresponding atom was determined. Finally, a matrix correction using the turbo quantum method was performed to convert the momentum numbers into mass fractions.

The resulting average values of the measured concentrations in ppm with the respective standard deviations were used for material characterization. The elemental composition of the samples can be compared to the acceptance criteria (legal framework requirements or defined by standards) for each use case. For example, the concentrations of halogens like Br can be used as an indication of the content in halogenated flame retardants. Regarding the model waste, the measured Br-content was used to calculate the concentration of TBBPA in the model waste sample on the assumption that all Br can be attributed to TBBPA.

#### 2.4.4. Gel Permeation Chromatography (GPC)

GPC analysis was used for the determination of the average molecular weights and molecular weight distribution of model waste samples (ABS-rich) from the different homogenization pathways. Experiments were performed with the use of an Agilent 1100 G1312A (Agilent Technologies Inc., Santa Clara, CA, USA) instrument, equipped with the columns PLgel 10 µm MIXED-B and a DRI 1100 G1362A. Model waste pellets were dissolved in THF at a 2 mg/mL concentration. Elution was carried out with THF at a flow rate of 1 mL/min. The calibration of the instrument was carried out with a polystyrene standard solution (580–6,870,000 g/mol; Polymer Laboratories, Long Beach, CA, USA).

#### 2.4.5. Melt Flow Rate (MFR)

MFR (g/10 min) was measured at 230 °C under 2.16 kg, according to the ISO 1133 standard [28,29], using a Dynisco model 4004 capillary rheometer (Dynisco LLC, Franklin, MA, USA) for the sorted PP-rich waste (PP-RE). For the model waste (which is ABS-rich), MFR was measured at 220 °C under 10 kg, according to the ISO 19062 standard [30] for ABS materials, using a Zwick Roell MFlow extrusion plastometer (Zwick Roell, Ulm, Germany). In both cases, MFR was expressed in grams of polymer flowing per 10 min, according to Equation (1):

(1) $$MRR(230\;{}^{\circ}C,\;2.16\;kg\;or\;220\;{}^{\circ}C,\;10\;kg)=\frac{600}{t}m_{avg}$$

where *t* (s) is the time between consecutive cuts of the molten material (cut-off interval), and *m*_avg_ (g) is the average mass of the material extruded.

#### 2.4.6. Thermal Properties

For the PP-rich sorted waste stream (PP-RE), differential scanning calorimetry (DSC) measurements were performed in a Mettler DSC 1 STARe System, through a heating-cooling-heating cycle from 30 up to 300 °C under N_2_ flow (20 mL/min) using a rate of 10 °C/min. For the ABS-rich model waste samples, DSC experiments were performed in a TA Instruments Q200 apparatus (TA Instruments, New Castle, DE, USA), through a heating-cooling-heating cycle from 0 up to 350 °C under N_2_ flow (50 mL/min) using a constant temperature ramp rate of 10 °C/min for heating and 5 °C/min for cooling. The glass transition temperature (*T*_g_, °C) and melting point (*T*_m_, °C) were derived from the 2nd heating cycle.

Thermogravimetric analysis (TGA) was also conducted for the PP-rich PWS in a Mettler TGA/DSC 1 thermobalance (Mettler-Toledo International Inc., Columbus, OH, USA) with a heating rate of 10 °C/min from 30 to 800 °C under constant N_2_ flow (20 mL/min). For the ABS-rich model waste samples, TGA was performed in a TA Instruments Q500 device with a heating rate of 10 °C/min from 30 to 900 °C under constant N_2_ flow (10 mL/min). The onset decomposition temperature (*T*_d,5%_, °C) was defined as the temperature at 5% weight loss, the degradation temperature (*T*_d_, °C) was determined at the maximum rate of weight loss and the percentage residue (W, %) was taken in the 30–300 region (for the model waste samples) or at 800 °C (for the filled PWS, PP-RE).

#### 2.4.7. Mechanical Properties—Uniaxial Tensile Testing

Uniaxial tensile testing was conducted using an Instron 4466 universal testing machine equipped with a 10 kN load cell (Instron Co., Norwood, MA, USA) according to ISO 527-2. The specimens used were Type 1BA (prepared using an in-house injection molding), and before testing, all specimens were conditioned according to ISO 291, ensuring uniform temperature and humidity conditions to minimize environmental influences on the results. Testing was carried out at a constant crosshead speed of 5 mm/min. From the obtained stress-strain curves, key tensile properties were evaluated, including yield strength (*σ*_y_, MPa), modulus of elasticity (*E*, MPa), and strain at yield (*ε*_y_, %). For each material, 10 specimens were tested to ensure statistical reliability, and standard deviation (SD) and relative standard deviation (RSD, %) values were calculated to assess the variability of the measurements.

#### 2.4.8. X-Ray Micro-Computed Tomography (mCT)

Segments derived from the gauge region of selected Type 1BA tensile testing specimens were analyzed with X-ray micro-computed tomography in a SkyScan 1272 High Resolution Micro-CT system (Bruker micro-CT, Kontich, Belgium). The principle of m-CT relies on combining Χ-ray attenuation of multiple radiographs obtained at different angular locations, “reversing” transmission images, and superimposing pixel intensities into a single attenuation value that is registered for a voxel in space. This allows us to isolate and analyze the constituent phases within the sample volume that present varying densities and compositions. During data acquisition, the relative source-detector positioning was adjusted to obtain a scanning resolution of 3.00 μm pixel size. Source voltage and current were set at 55 kV and 160 μA, respectively, and an X-ray filter (Al, 0.25 mm thickness) was applied. Each sample rotated over 360 degrees with a fixed rotation step of 0.250 degrees, and a shadow (i.e., transmission) image was acquired at each angular position at 2541 ms exposure. The obtained shadow projections were used for the reconstruction of grayscale slices through each sample, generated using NRecon reconstruction software (v1.7.0.4 by Bruker micro-CT), by implementing the Feldkamp algorithm. The original grayscale slices were processed in CT-Analyser (v1.18.4 by Bruker micro-CT) for isolation and segmentation of constituent phases. Reconstructed results were processed in DataViewer software (version 1.5.6.2 by Bruker micro-CT) and CTvox software (version 3.3.1 by Bruker micro-CT) for volume rendering and 2D/3D visualization.

## 3. Results and Discussion

### 3.1. Challenges of Sampling and Sample Preparation in Plastics Recycling—PP Rich Waste

A real PP waste coming from refrigerators (PP-RE) was sorted and thoroughly characterized as reference, in order to reveal and emphasize the challenges of sampling and sample preparation in plastics recycling. Firstly, the polymeric composition and contamination levels were determined by means of NIR spectroscopy (obtained in the 10 kg scale). A high percentage of polyolefins was revealed, i.e., PP (98% wt.) and PE (1.45% wt.), while other polymeric contaminants at lower concentrations involved styrenic polymers (ABS 0.12%, PS 0.01% wt.), PVC (0.38% wt.), and PA (0.04% wt.).

Randomly picking five flakes (Figure S1a) and analyzing them via ATR-FTIR (Figure S2) and DSC (Figure S3) verified the presence of different polymer types and also emphasized the heterogeneity of the waste. More specifically, gray, white, and yellowish flakes can be matched to PP by manually inspecting the ATR-FTIR spectra in combination with the OPUS Software^®^ quick compare setup with the spectra database, while PVC (brown and blue flakes), ABS (black flakes), and PC (red flakes) were also recognized. The same conclusions were drawn by the DSC analysis, in which gray flakes demonstrated a sharp melt crystallization peak at 127 °C (Figure S3a), as well as endotherm peaks at approximately 163–165 °C during the 2nd heating cycle (Figure S3b), which can safely point towards PP with a calculated degree of mass fraction crystallinity (x_c_) equal to 24% (Δ*H*_0_ = 207 J/g) [31]. The other ones, being amorphous (ABS, PC, PVC, etc.), exhibited only glass transitions, approximately 85 °C and 107 °C for PVC and ABS, respectively.

XRF (performed on a 3–5 g melt-prepared pellet) and TGA are useful for evaluating the presence of inorganic fillers, additives, or char formation, which may impact reprocessing and thus recycling quality. XRF spectroscopy (Table 2) showed high percentage (and deviations) of inorganic substances and metals (Si, Ca, Ti) in PP-RE deriving from additives that are usually used in EEE polymers: the presence of silicon (Si) can provide information about glass fibers content and/or talcum, calcium (Ca) can be related to calcite (CaCO_3_) used as a filler, titanium (Ti) often relates to the whitening pigment TiO_2_, antimony (Sb) can be present as Sb_2_O_3_, which has a flame-retardant synergy, while hazardous heavy metals only could be measured in minor concentrations.

**Figure 2:**
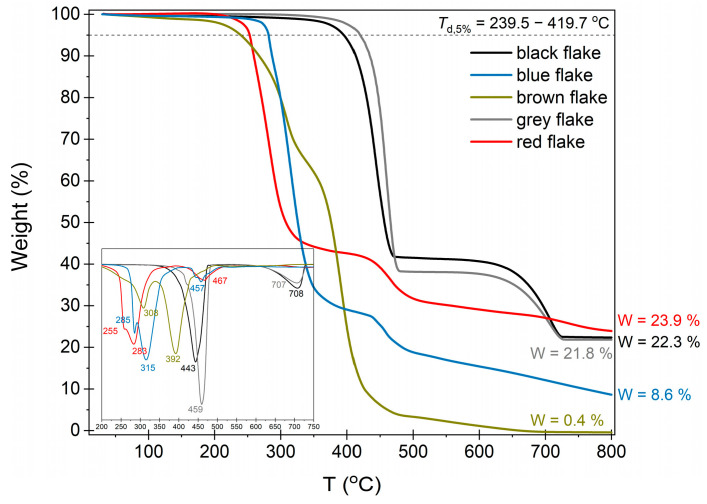
TGA curves of five different colored flakes of sorted PP-rich EEE waste stream from refrigerators (PP-RE). The dash line represents the 95% of mass corresponding to *T*_d,5%_.

Additionally, TGA curves in Figure 2 showed that, depending on the flake analyzed, a range of final residues (W = 0.4–30.3% at 800 °C) and one to three degradation/decomposition steps over a broad temperature window were identified, meaning that some polymer types were filled and some not—containing also different loadings and possibly different types of fillers—which unavoidably led to high variability within the replicates. Apart from the polymer type effect on sorted stream thermal stability, mineral fillers and especially metal oxides, depending on their chemical nature and interaction with the polymer matrix they are used in, may catalyze or accelerate the degradation of a material, due to their reactivity or by creating sites that facilitate degradation, which can be manifested in the *T*_d,5%_ and *T*_d_ values.

Indeed, the onset of thermal degradation (*T*_d,5%_) varied significantly depending on the flake, from low temperatures of 240 up to 420 °C, signifying differences in the thermal stability of the input material, which can have a huge impact upon (re)processing (extrusion or injection). The same picture was observed for the *T*_d_ values, which roughly ranged from 280 to 470 °C. This already clearly demonstrates that sampling on the flake level is not adequate. Further manifestation of this heterogeneity could also be observed visually (Figure S1b) from the different colors (different polymer phases) on an injection-molded sample’s surface, as well as from MFR measurements that exhibited significant variations between replicates.

In particular, MFR—which is directly related to melt flow behavior and processability—presented substantial variations, i.e., from 7.5 to 9.8 g/10 min, among the five replicates (Table 3). Apart from inter-replicate differences, there are also intra-replicate ones that are evident in the high standard deviation (SD) and relative values (RSD = 5–14%), in contrast to virgin materials, where 0.5–2% RSD values are expected [28,29]. Also, according to the ISO 1133 standard [28], the difference between the maximum and minimum values in each data set should not exceed 15% of its average; from the five replicates herein, only Replicate 3 is barely within the guidelines (1.2 in Table 3), with the other ones (in a red font in Table 3) being far away from the acceptable margins. Consequently, 8.2 ± 0.4 g/10 min (5%) is the only acceptable MFR value, but the inconsistency and low precision of the other four replicates raise issues regarding the representation that one replicate provides of the lot, considering the typical high input mass of real plastic wastes. Practically, homogenization issues render the MFR determination not feasible for assessing the processability of the input material.

**Figure 3:**
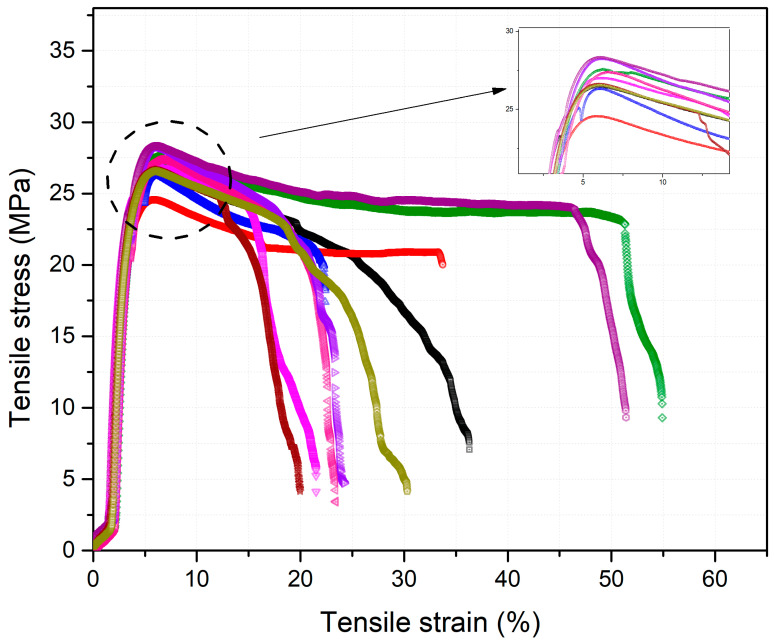
Tensile stress-strain curves of ten replicates of sorted PP-rich EEE waste stream from refrigerators (PP-RE).

The PP-rich sorted waste stream was also characterized via tensile testing; the material heterogeneity was visually apparent with multiple color variations in the prepared dumbbell 1BA specimens (Figure S1b), suggesting different phases or regions with distinct compositions. The stress-strain curves of ten specimens of PP-RE material in Figure 3 revealed significant variability in tensile properties, which suggests a lack of consistency in the material’s composition and structure. The presence of distinct phases within the specimen and discontinuities acted as stress concentrators, leading to premature failure and reduced mechanical performance.

Furthermore, the multiple phases led to varied mechanical responses under tensile stress: some regions may be more ductile, while others may be brittle, causing unpredictable failure patterns. This heterogeneity leads to increased variation, as can be observed by the increased RSD % in yield properties, making the material unreliable for applications where mechanical uniformity is essential (Table 4).

**Figure 4:**
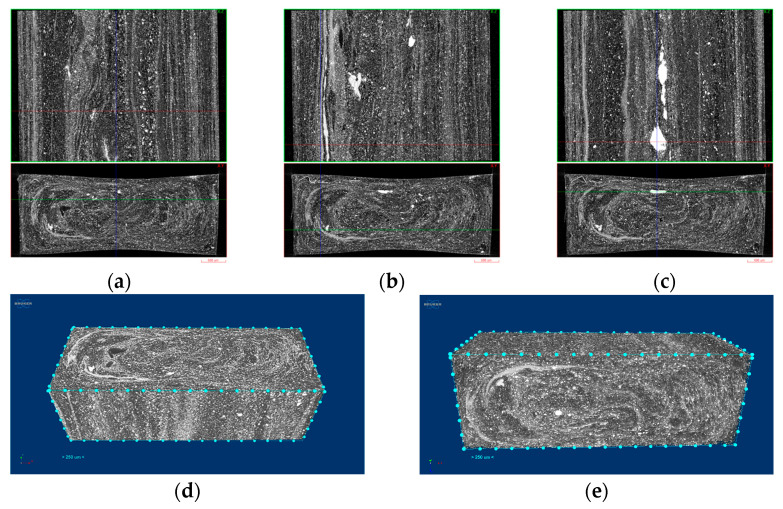
(**a**–**c**) Representative XZ and XY cross sections of the reconstructed grayscale slices of the samples (DataViewer software v.1.2.5.7, Bruker microCT); scale bar: 600 μm, (**d**,**e**) 3D visualization of sample volume (CTvox, software v3.3.1, Bruker microCT). Segmentation of dispersed particles and areas in the polymer matrix with different X-ray attenuation levels are highlighted in green and light blue, respectively.

Finally, the materials heterogeneity was also confirmed by X-ray micro-computed tomography. Reconstructed results obtained by mCT were initially visualized as a set of orthogonal slices crossed at selected points of the sample volume. Phases with high X-ray attenuation are highlighted towards white in grayscale, and the surrounding polymer matrix is depicted in darker gray, while air inclusions and ambient air are depicted as black (Figure 4). A dispersed phase consisting of particles within a 10–20 μm range (towards white in grayscale) is observed, possibly associated with inorganic fillers or metals, which present higher X-ray absorption than the surrounding polymer matrix, also in agreement with XRF analysis. Distinct high X-ray absorption particles of larger sizes (100–200 μm) are also observed, possibly associated with contaminants. Additionally, the polymer matrix itself presents different X-ray attenuation levels, indicating an inhomogeneous mixture possibly originating from an insufficient blending of different colored flakes of sorted PP-rich EEE waste stream, as different types of pigments (e.g., metal oxides vs. carbon black) interact differently with X-rays.

The aforementioned results clearly show that plastic waste analysis is highly dependent on the choice of individual flake(s), and analyzing a vast number of flakes (so that their sampled distributions *s_i_* will be more representative of the lot) is impossibly time-consuming, impractical, and cost-ineffective. Therefore, there is a need for appropriate homogenization and sampling strategies prior to analyzing the PWS, which can generate results accurate and representative enough to describe the contents and the safety of the bulk, while being practical, time- and cost-effective. Homogenization strategies discussed hereafter aim to intensify the efforts for identification and precise quantification of different polymer types and non-polymeric contaminants, which is crucial for recycling purposes in terms of (i) assessing the waste degradation extent, (ii) choosing suitable processing techniques and their temperature window, and (iii) selecting the appropriate recycling/upcycling strategy.

### 3.2. Theoretical Approach to Evaluate Sample Preparation Techniques—ABS Rich Waste

The model waste approach served herein as a blueprint to use analytical methods typically employed in plastics recycling and to understand how to expect their performance in terms of accuracy and precision for plastics waste characterization. In an initial attempt to estimate the influence of sample particle size and sample quantities suitable for different analytical methods, theoretical calculations were employed. The assumptions of uniform flake mass and uniform particle size after grinding were intentionally adopted to construct a simplified and transparent theoretical framework, rather than to reproduce the full complexity of industrial waste streams. Accordingly, the model waste composition was assumed, as shown in Table S1, and first, the samples’ morphology and size were considered similar to a real sorted waste stream material, like in Figure S1a: 1 kg consists of almost equally sized flakes of 0.05 g per flake, resulting in the theoretical number of 20,000 total flakes.

As a first step, it was assumed that 10 samples of 3 g each (60 flakes in 3 g) were drawn, and the individual flakes were theoretically analyzed indicatively by ATR-FTIR, as mentioned above for the different colored flakes of the sorted PP-rich waste, to determine the polymer composition of the bulk material. The selection of 10 samples was chosen as a practical compromise that allows estimation of inter-sample variability and relative standard deviation in conventional lab analysis methods. Replicate numbers of this order are commonly employed in analytical practice to characterize sampling uncertainty in heterogeneous materials, while remaining viable in terms of time, material availability, and analytical effort.

**Figure 5:**
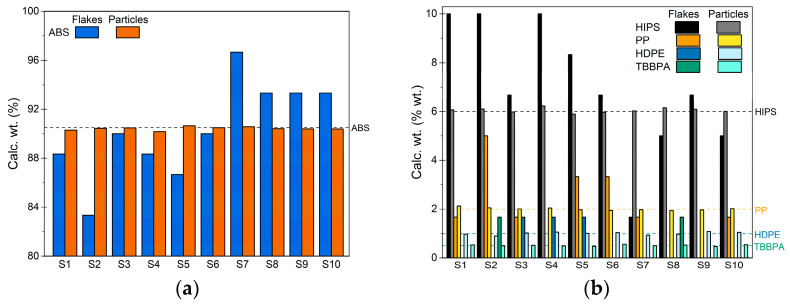
Calculated polymer composition of 10 theoretical samples (sample mass = 3 g, 60 flakes) of sorted ABS-rich waste stream (model waste ABS nominal composition = 90.95% wt.) for flakes vs. particles. (**a**) ABS and (**b**) HIPS, PP, HDPE, and TBBPA calculated content.

In Excel, cells were assigned to each polymer (equal to their share in the mixture), and by implementing a simple Monte-Carlo simulation, a cell-polymer is randomly picked from the lot, and each component content can be calculated. It was readily found that the dominant polymer, ABS, was detected in all 10 samples (Figure 5a), but could not be quantified with sufficient accuracy since it accounted for 90.3 ± 3.9% wt. on average. (Table S1, RSD = 4%), while the nominal value was 90.95% wt. The ABS content in individual samples, however, was partially significantly underestimated (<84% in S2) or overestimated (>96% in S7). Polymers at lower concentrations, like PP (2.0% share) and HDPE (1.0% share), were not present in every sample (Figure 5b) and might not be detected at all when fewer samples are analyzed; PP and HDPE reached average values of 1.8 ± 1.7% wt. (RSD = 94%) and 0.5 ± 0.8% wt. (RSD = 160%), which practically renders PP and PE not reliably detected when the sorted flakes are directly analyzed in this scheme. Similarly, TBBPA contaminant could not be reliably detected since the calculated content was found at 0.3 ± 0.7% wt. and a huge RSD value (>200%).

A way to increase accuracy and precision is to decrease particle size before sampling [32], permitting better homogeneity, more statistically representative subsamples for analysis, and improved distribution of fillers/additives. Theoretical grinding of the flakes to smaller particles was thus implemented: 1 kg of the bulk sorted material was computationally “ground/milled”, mimicking, e.g., a ring sieve mill, by uniform splitting of the initial lots to a (uniform) simulated final particle size of 750 µm. This leads to 400,000 particles from 20,000 initial flakes. Each component content, along with the non-polymeric contaminant (TBBPA), was again calculated based on 10 theoretical samples of 3 g each (Table S1): following “grinding”, the average contents for all polymers and TBBPA were now found much closer to their nominal values (Figure 5b), and RSDs were significantly decreased compared to the flakes considered earlier. As expected, grinding/milling increased the homogeneity significantly, reduced the “shot noise” sampling error of large flakes, and allowed better analytical results.

**Figure 6:**
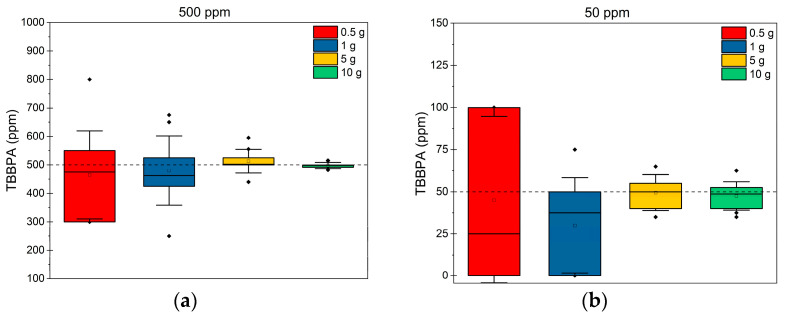
Calculated TBBPA content of ten theoretical samples for each sample mass (0.5–10 g) in the ground sorted ABS-rich waste stream for nominal concentrations (shown as dash lines) of (**a**) 500 ppm and (**b**) 50 ppm. Solid line represents the median, hollow squares and diamond symbols represent the mean value and outliers, respectively.

Considering that many analytical methods are limited to small, required sample amounts (<10 g), and that sample preparation and analysis cannot be scaled up easily, the accuracy and precision of detecting the presence of lower-concentration components were further correlated to different sample sizes. On the basis of the 400,000 virtual particles after grinding, the average TBBPA contents for the nominal concentration of 500 ppm (0.05 wt. %) were calculated for different sample amounts (0.5–10 g), a range that is typically used on lab-scale analytical techniques. The results are presented as boxplots in Figure 6: the box embraces the second and the third quartile (quartiles q2–q3), which means that the middle 50% of the data is captured within the box, indicating the width of the distribution of all ten theoretical samples. As a result, the narrower the box, the closer the individual values are and thus, the more homogenous and reproducible the replicates. As can be seen in Figure 6a for the nominal TBBPA content of 500 ppm, 0.5 g of sample was found sufficient to detect it in all ground samples, even though its actual concentration was not determined accurately (q2 to q3 ranged from 300 to 550 ppm). As expected, analysis of a larger sample amount yielded more accurately quantified results: a sample amount of 10 g presented almost perfect accuracy and precision (q2 to q3 from 492 to 499 ppm), while in the case of 1 g, which is more commonly used as a typical sample mass for many analytical techniques, the values showed a higher variance of more than ±200 ppm (q2 to q3 ranged from 425 to 525 ppm).

On the other hand, when decreasing the nominal TBBPA content to 50 ppm (Figure 6b), the theoretical calculations revealed that the flame retardant may not be detected in all samples when 1 or 0.5 g were considered (q2 to q3 ranged from 0 to 50 and 99 ppm, respectively). A higher amount of sample is needed (5 or 10 g) to detect TBBPA presence in all ten samples; however, even such an amount is insufficient for precise quantification since calculations presented respective variance (q2 to q3 ranged from 40 to 53 ppm).

These theoretical results for the precise and accurate detection and/or quantification of non-polymeric contaminants can also be transferred to an industrial scale, showing the challenges that the plastics recycling industry confronts today, especially since more and more strict regulatory standards are set. Even for the case of 500 ppm contaminant, a minimum of 1% (10 g out of 1 kg) of ground sorted material needs to be analyzed to obtain statistically robust assessments of contamination levels. Within the simulated setting, assuming homogeneous flake sizes and sizes of ground particles as described above, this sample amount counts for 200 kg in a typical batch of 20 tons, implying a not-applicable large number of samples, as well as significant costs for sample preparation (e.g., grinding) and for analytics. The theoretical approach herein, therefore, sets a framework to balance practical feasibility, analytically available tools/methods, and a representative sample preparation strategy. These aspects are experimentally investigated in the next sections of the current paper, where two more homogenization options were assessed: extrusion and injection molding, both combined with cryogenic grinding. Note that in any real-world implementation, flake and ground particles will always have size distributions, which will also be different for different species. Such effects could also be treated by more involved simulation or computational treatment, but require many assumptions and data that seem hard or impossible to experimentally obtain.

### 3.3. Experimental Approach to Evaluate Sample Preparation Techniques—ABS Rich Waste

#### 3.3.1. Evaluate Possible Thermomechanical Degradation of Samples

The homogenization conditions applied in this work were selected to remain within industrially relevant processing windows for styrenic and polyolefin-based materials. However, grinding, extrusion, and injection molding as sample preparation strategies stress the polymers mechanically and thermally, which can lead to changes in the molecular weight and molecular weight distribution, hence altering the sample and pointing out the need to monitor such changes during sample preparation. Therefore, to ensure the accuracy of the model waste study, it was important to evaluate any potential degradation during blending/homogenization and sample preparation. Given that extrusion can induce polymer chain scission due to elevated temperatures, on the basis of the herein examined model waste, the molecular weight distribution of ABS was assessed using GPC. Consistent values for both weight-average (${\bar{M}}_{w}$) and number-average molecular weight (${\bar{M}}_{n}$) were found when comparing the different model waste samples to virgin ABS (Table 5), revealing no significant polymer molecular weight decrease under the conditions of the herein examined blending/homogenization techniques.

**Scheme 1:**
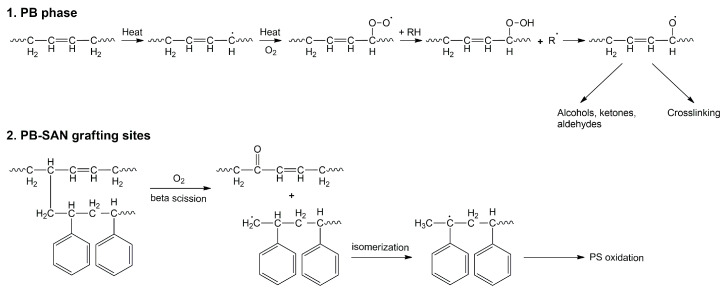
Degradation mechanism of ABS (adapted from reference [23]).

Additionally, changes in the chemical structures of the polymers due to any degradation were sought in the relevant ATR-FTIR spectra. Being ABS-rich, these chemical changes would be manifested in terms of hydroxyls (–OH at 3180–3800 cm^−1^) and carbonyls (–C=O at 1680–1800 cm^−1^) generation, as well as in the reduction of the double bond groups from the polybutadiene (PB) phase, corresponding to the peaks of *trans*-1,4 (965 cm^−1^) and *vinyl*-1,2 (911 cm^−1^) groups. Degradation is primarily initiated in the PB phase, which is selectively attacked during the initial stages, leading to the consumption of the unsaturated sites and subsequent formation of oxidized moieties. Further reactions, involving free radicals and hydroperoxides, may lead to the formation of alcohols, ketones, and aldehydes (hence the generation of –OH and –C=O), as well as stable crosslinks (Scheme 1) [23,33,34].

**Figure 7:**
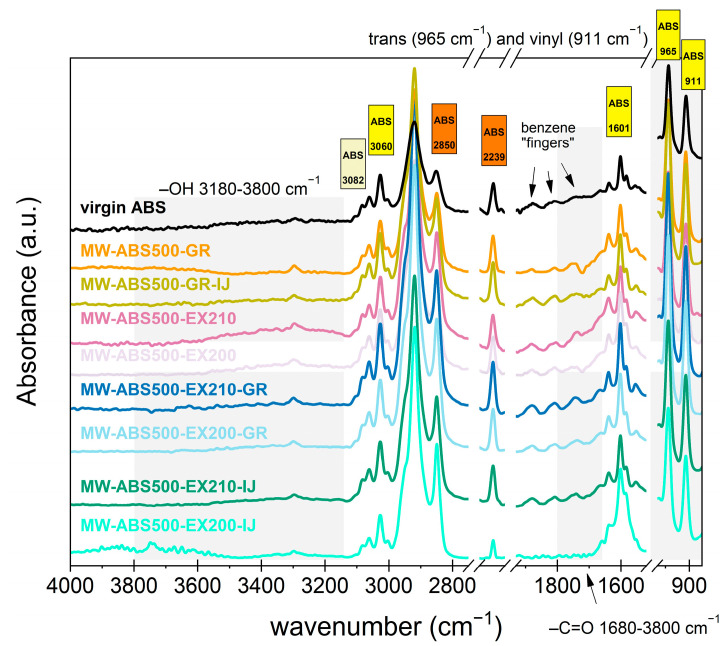
ATR-FTIR spectra of virgin ABS and model waste samples prepared via different blending/homogenization techniques in the 880–4000 cm^−1^ region.

As seen in Figure 7, no evident increase was observed in the −OH region that could point out any degradation products. The same goes for the −C=O region, since the low-intensity pattern of three evenly spaced bands in the 1700–1920 cm^−1^, labeled as benzene “fingers”, is not associated with degradation, but it is a common feature of mono-substituted benzene rings. In addition, *trans*-1,4 and *vinyl*-1,2 peaks (which are already present in ABS and HIPS) did not exhibit any significant reduction in their intensity. Therefore, it is safe to assume that neither the combination of sample preparation strategies (e.g., cryogenic grinding following extrusion) nor the higher extrusion temperature profile had a significant adverse effect upon the chemical structure of the polymers used in the model waste composition. 

#### 3.3.2. Evaluate the Efficiency to Define Content of Undesirable Additives (Non-Polymeric Contaminant)—TBBPA Content

**Figure 8:**
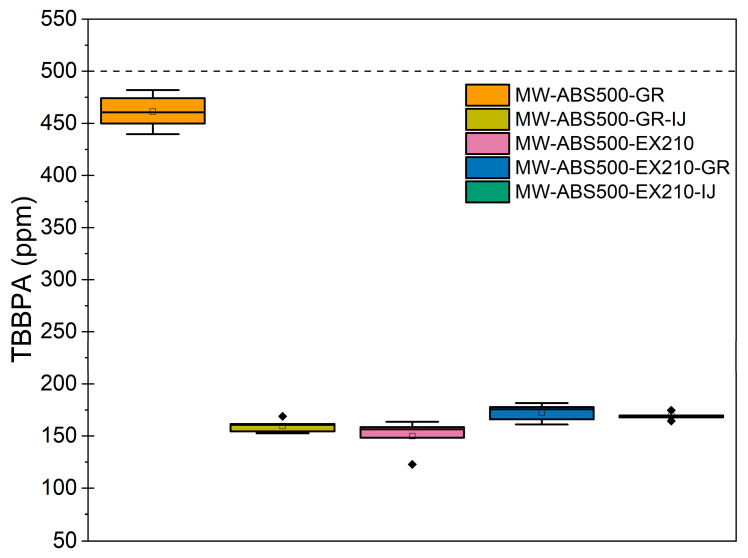
Boxplot diagram of the analyzed TBBPA concentrations measured by HPLC-MS in the model waste samples prepared via different blending/homogenization techniques. Solid line represents the median, hollow squares and diamond symbols represent the mean value and outliers, respectively.

The accurate quantification of legacy additives, such as brominated FRs like TBBPA, in recycled plastics drives the need to develop a homogenization approach that yields the lowest deviation between replicates, as well as proximity to the actual concentration values. In view of that, HPLC-MS-based quantification of TBBPA for the nominal concentration at 500 ppm was conducted for the examined homogenization methods. Five replicates were performed on the different model waste samples, and the results, hereafter, are presented as boxplots. When comparing the different homogenization methods in terms of TBBPA measurements (Figure 8) for a 1 g sample size, the flame retardant was detected in all five replicates. Its distribution was found to be the broadest for the sample that was only ground (MW-ABS500-GR), since q2 to q3 ranged from 450 to 474 ppm TBBPA, with an average concentration of 461 ± 17 ppm. Applying only extrusion (MW-ABS500-EX210) resulted in a slightly narrower distribution (q2 to q3 ranged from 148 to 158 ppm), while combining it with a subsequent grinding step (MW-ABS500-EX210-GR) or with injection molding (MW-ABS500-EX210-IJ) resulted in much narrower distributions (166 to 178 and 168 to 170 ppm, respectively). This is also depicted in the low RSD values (2–5%) for the average TBBPA content when adopting the combined homogenization strategies. To determine whether these deviations are not due to instrumentation error, a single sample was also analyzed five times, quantifying one analyte: the RSD (%) across these measurements was approximately 1%, indicating that the observed effects for the model waste samples are attributable to inhomogeneities, and any homogenization attempt can be reliably evaluated when RSD > 1%.

For the homogenization methods that required the material to be heated and/or molten (extrusion, injection), however, a significant decrease in TBBPA content was monitored via HPLC-MS, from roughly 461 ± 17 ppm (for MW-ABS500-GR) to as low as 150 ± 16 ppm (MW-ABS500-EX210), suggesting TBBPA thermal degradation due to high temperatures used for extrusion and/or injection molding. In other words, even though the ground/milled samples showed the broadest value distribution, when the objective is to detect and quantify small molecules like additives, this sample homogenization method seems favorable to avoid all risks for any content underestimation due to any volatility or thermal instability of such species to be detected.

**Figure 9:**
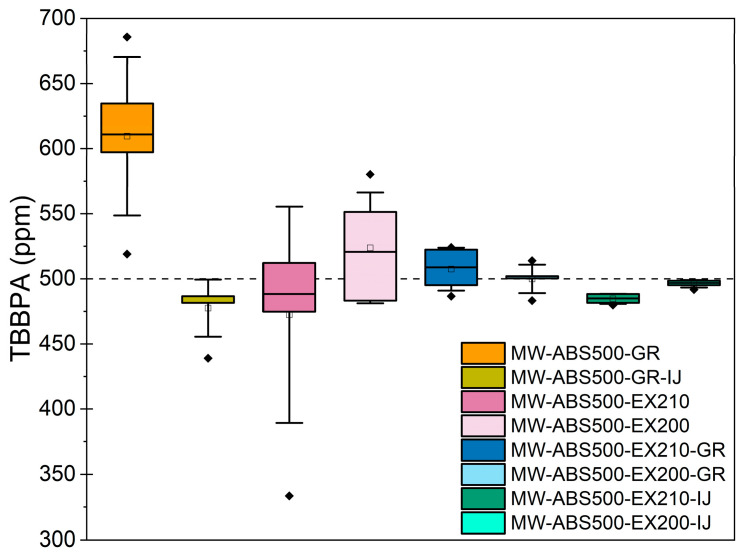
Boxplot diagram of the calculated TBBPA concentrations by using Br content measured by XRF spectroscopy in the model waste samples prepared via different blending/homogenization techniques. Solid line represents the median, hollow squares and diamond symbols represent the mean value and outliers, respectively.

In Figure 9, a boxplot diagram of calculated TBBPA concentrations in different model waste samples is based on the XRF quantification of the elemental (Br) composition, considering that TBBPA has a mass share of Br equal to 58.8% wt. The XRF results (sample size 3–5 g) assume that all quantified Br is attributed to TBBPA, and therefore may overestimate TBBPA content, particularly if other Br-containing substances or degradation products were present or formed due to high temperatures during the homogenization. For this reason, XRF results were not interpreted as absolute TBBPA concentrations, but valorized comparatively to assess distribution and homogenization efficiency. Accordingly, it was found that XRF trends are in line with HPLC-MS findings; XRF, being a relative analytical technique (calibrant-dependent), yielded TBBPA values higher than 500 ppm (especially for the MW-ABS500-GR), while HPLC-MS in general presented absolute, more accurate results. Still, by comparing the trends and evolution of TBBPA concentration with different blending/homogenization, similar conclusions between the two analytical techniques can be drawn. In particular, the average values, distribution of the five measured replicates, and the quartiles q2 to q3 of the TBBPA content spread the most for the samples MW-ABS500-GR, MW-ABS500-EX210, and MW-ABS500-EX200, which were only ground or extruded at 210 °C or 200 °C, respectively, for homogenization purposes. The average TBBPA contents presented a range from 472 to 610 ppm, which differed significantly from the nominal concentration of 500 ppm. On the other hand, when combining the homogenization steps, i.e., grinding and injection, extrusion and grinding, as well as extrusion and injection, showed a significantly narrower distribution of the measured Br-content/calculated TBBPA-content. Whereas for MW-ABS500-GR-IJ (grinding and injection), the quartiles q2 to q3 range from 460 to 487 ppm TBBPA, with an average concentration of 477 ppm, the distribution of the measured TBBPA concentrations is even narrower for MW-ABS500-EX210-GR and MW-ABS500-EX200-GR with average concentrations of 507 and 500 ppm, respectively. When extrusion was followed by injection, even higher homogenization efficiency was achieved: the quartiles q2 to q3 ranged only from 481 to 488 ppm for MW-ABS500-EX210-IJ and from 497 to 499 ppm for MW-ABS500-EX200-IJ, with respective average concentrations at 485 and 496 ppm TBBPA deviating slightly from the nominal concentration.

**Figure 10:**
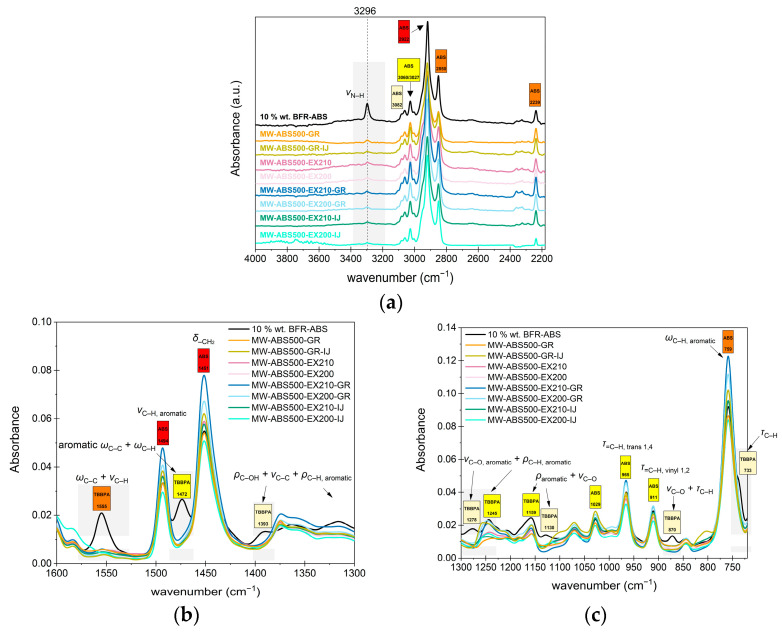
ATR-FTIR spectra of 10% wt. BFR-ABS and model waste samples prepared via different blending/homogenization techniques containing 500 ppm TBBPA in different IR regions: (**a**) the 2200–4000 cm^−1^, (**b**) 1300–1600 cm^−1^, and (**c**) 700–1300 cm^−1^ regions.

The detection of TBBPA was also attempted by means of ATR-FTIR spectroscopy (sample size 200 mg/film). Starting with the MWIR region (Table S2), TBBPA was not visible in any of the model waste samples, irrespective of the homogenization approach; that was also the case even for the high concentration of TBBPA (10% wt. BFR-ABS), which bodes well with the literature [35]. TBBPA is rather elusive in this region, with the 3515 and 3475 cm^−1^ peaks (O−H stretching vibration attached to the aromatic rings) expected to appear only at even higher loadings (e.g., 34% wt.) [35]. Interestingly, a peak located at 3296 cm^−1^ demonstrated relatively high absorbance in the 10% wt. BFR-ABS spectra, but milder in the model waste samples, as clearly seen in Figure 10a. Although this peak can be falsely attributed to a shifted O–H stretching vibration peak, it is instead associated with typical lubricants used in ABS processing, like ethylene bis-stearamide (EBS), owing to the N–H stretching vibration of secondary amide groups. In the model waste samples, this absorbance is reduced due to its possible loss, especially at temperatures > 200 °C, as was the case herein during extrusion and/or injection molding processes.

At the 1600–1300 cm^−1^ region (Figure 10b), a small but sharp peak of TBBPA appeared at 1555 cm^−1^, which corresponds to the out-of-plane bending of C−C and stretching vibration of C−H in the 10% wt. BFR-ABS. In addition, a smaller one at 1472 cm^−1^, referring to the out-of-plane bending of C−C and C−H of the TBBPA aromatic ring, inserts itself between the peaks at 1494 cm^−1^ (stretching vibration of C−H of the ABS aromatic ring) and 1451 cm^−1^ (scissoring mode of −CH_2_ present in ABS). The aforementioned TBBPA peaks are very discernible for the 10% wt. BFR-ABS formulation, but not in any of the model waste samples, regardless of the homogenization approach.

In the LWIR (Table S3), some of the TBBPA characteristic peaks were identified at 1393 and 1317 cm^−1^, which were attributed to (C–)O–H bending, C–C stretching, and aromatic C–H deformation, respectively. Also, shifts to higher wavenumbers were observed for the anticipated 1272 and 1238 cm^−1^ peaks of TBBPA (C–O stretching in aromatic ring, contribution from aromatic C–H in-plane bending), towards 1278 and 1245 cm^−1^, respectively, for the 10% wt. BFR-ABS due to interactions with the polymer matrix (Figure 10c). On the other hand, the 1226 and 1197 cm^−1^ peaks were not detectable, which is anticipated for TBBPA loadings < 17% wt. [35]. The 1173 cm^−1^ peak of C–O–C stretching, although usually serves as a TBBPA visible marker [35], was similarly not evident even at the 10% wt. BFR-ABS sample, while a broad peak centered at 1159 cm^−1^ (1157 cm^−1^ for pure TBBPA) and a “hard-to-see” one at 1130 cm^−1^ (1132 cm^−1^ for TBBPA) [35,36] appeared. The 940, 887, 780, 648, and 616 cm^−1^ peaks were not observable, while the 867 cm^−1^ one (overlap of asymmetric stretching of C−O, out-of-plane bending of C−H in TBBPA) was slightly shifted to 871 cm^−1^, but of small intensity [36]. Moreover, the TBBPA peak of C−H twisting vibration expected at 731 cm^−1^ is moderately visible in the 10% wt. BFR-ABS, as it introduces itself, as a shoulder at 733 cm^−1^ between the two aromatic C−H and =CH wagging and scissoring peaks of ABS centered at 759 and 698 cm^−1^. Ultimately, there were peaks of TBBPA that were clearly observable in the 10% wt. BFR-ABS, however, none could be identified in any of the different sampling approaches, meaning that such concentrations (500 ppm) were below the detection limit of ATR-FTIR spectroscopy.

**Figure 11:**
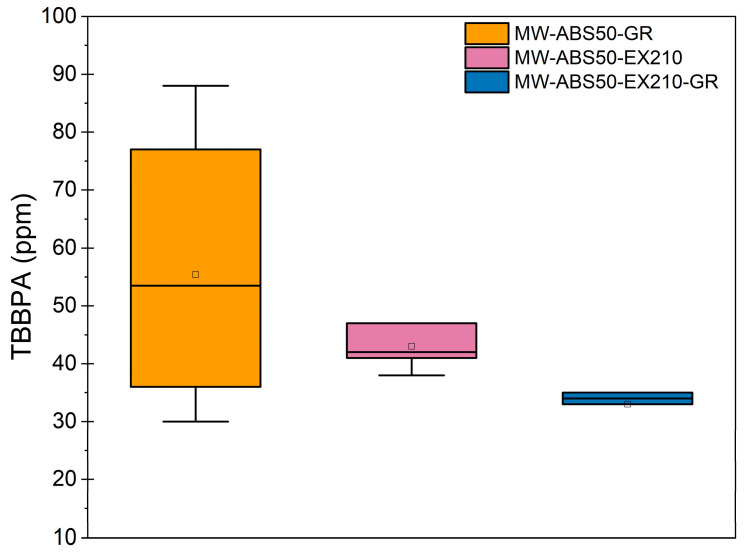
Boxplot diagram of the analyzed TBBPA concentrations measured by HPLC-MS in the model waste samples prepared with a nominal concentration of 50 ppm TBBPA.

HPLC-MS measurements were also performed for the 50 ppm model waste samples using the two main homogenization techniques (grinding and extrusion), as well as their combination (extrusion and grinding) (Figure 11). The main observation was similar to the 500 ppm samples: grinding alone yields the broadest distribution, since q2 to q3 ranged from 36 to 77 ppm TBBPA, with an average concentration of 55 ppm (±20 ppm, RSD = 36%), since even a small particle containing less or more TBBPA might significantly influence the results in the ppm range. Samples that underwent only extrusion, as well as those that were extruded and subsequently ground, displayed narrower distributions: q2 to q3 ranged from 41 to 47 ppm TBBPA for the extruded sample (43 ± 4 ppm), and q2 to q3 ranged from 33 to 35 ppm TBBPA for the extruded-ground sample (33 ± 3 ppm, RSD = 9%). These experimental findings align with the theoretical calculations, revealing a consistency between the measurements and the predictions, e.g., the theoretical model estimated ground material values between 0 and 75 ppm for a 1 g sample (Figure 6), which was also the experimentally found ppm range. Consequently, this simplified modeling approach, assuming uniform particle size and no variations in particle size distribution, can be reliable since deviations due to particle size heterogeneity may be minimal under certain conditions.

#### 3.3.3. Evaluate the Efficiency to Define Content of Polymeric Contaminants—HIPS, PP, HDPE Contents

The analysis was also directed at identifying the polymeric contaminants of the model waste (if we consider ABS as the matrix), namely HIPS (6% wt.), PP (2% wt.), and HDPE (1% wt.). Additives and polymeric contaminants can influence thermal properties because the former can act as plasticizers (lubricants) by reducing intermolecular forces between polymer chains and the latter as nucleating agents, which may alter the crystallization behavior. This effect is more pronounced in amorphous polymers (like ABS, HIPS) or in the amorphous fraction of semi-crystalline polymers (like PP and HDPE), where chain mobility is more affected [37,38,39,40]. DSC may not directly detect their presence, but shifts in characteristic temperatures and transitions may indicate interactions with the polymer matrix.

Overall, all DSC (sample size *ca*. 10 mg) thermograms were very reproducible (Table S4, RSD < 3%) except for the MW-ABS500-GR sample (ground/milled), which seemed to be a more inhomogeneous material in terms of melting properties characterization. The glass transition, approximately 103–107 °C, was associated with a styrenic component (from ABS or HIPS), while above 107 °C, approximately 127–131 °C, there is an endotherm peak caused by reordering/relaxation movements just after the *T*_g_ of the ABS [41,42]. In this case, this peak also coincides with the melting temperature of HDPE (between 130 and 140 °C), and therefore, the *T*_m_ of the PE fractions overlap. The second endothermic peak, at ca. 163–166 °C, is associated with the PP fraction. The only remarkable difference among the different blending/homogenization techniques observed for the MW-ABS500-GR sample, which interestingly exhibited the lowest *T*_g_s (94.8 ± 3.1 °C, RSD = 3.3%) and *T*_2_s (162.8 ± 0.5 °C, RSD = 0.3%), possibly due to some plasticization effects from the lubricant, in accordance with MFR (Section 3.3.4). When injection molding was applied following grinding (MW-ABS500-GR-IJ), the measured *T*_g_ increased roughly by 10 °C, potentially due to shear mixing and/or partial volatilization of the lubricant.

Regarding ATR-FTIR, the spectra of films were compared (Tables S5 and S6), and characteristic peak positions were found to be reproducible within a ±2 cm^−1^ margin. In the MWIR, ABS can be distinguished from the other three polymers in all model waste samples due to the C≡N stretching vibration, which generates a moderate but sharp signal at 2237 cm^−1^. Also, in LWIR, ABS exhibits strong peaks located at 757 and 698 cm^−1^. On the other hand, HIPS cannot be easily distinguished from ABS due to the simultaneous presence of the styrene and polybutadiene (PB) phase in both polymers. More specifically, in the MWIR (Table S5), the C−H stretching vibrations of the aromatic ring (at 3080, 3060, 3025, and 3000 cm^−1^), as well as the aliphatic ones (2920 and 2850 cm^−1^), are identical or in the vicinity of the ABS signals (±2 cm^−1^). Generally, ABS is loaded with more PB (more aliphatic C−H) than HIPS [43], ranging from 5 to 30% wt. compared to 6–15% wt. in HIPS, and thus, aliphatic signals are stronger than the aromatic ones (higher ratio aliphatic/aromatic) for ABS. As a result, the high loading of ABS overshadowed the presence of HIPS. For the LWIR regions, in principle, the aromatic C−H scissoring is centered at 693 and 749 cm^−1^ for HIPS and slightly shifted to approximately 698 and 757 cm^−1^ for ABS, due to the presence of acrylonitrile in the polymer chains that affects aromatic C−H vibrations (Table S6) [43,44]. Also, the PB phase has only a few signals, with only two being easily detectable at 965 cm^−1^ (=C−H deformation of PB *trans*-1,4) and 908 (HIPS), 910 cm^−1^ (ABS) (=C−H out-of-plane deformation of PB *vinyl*-1,2). Because of the higher PB loading of ABS, intensity differences in those peaks can help discriminate against ABS and HIPS samples, but it cannot identify one of them inside the other in polymer blends, e.g., in our model waste.

PP can be distinguished from the other three polymers by using the 2949 cm^−1^ peak (asymmetric stretching of −CH_3_), which appeared as a shoulder. Also, apart from the C−H asymmetric and symmetric stretching vibrations of −CH_2_ (2916 and 2840 cm^−1^) and −CH_3_ (2949 and 2867 cm^−1^), PP displayed a hard-to-see, but distinct peak (pale yellow color) at 2723 cm^−1^ in the MWIR. In the LWIR, PP exhibited several moderate to weak signals between 800 and 1170 cm^−1^, corresponding to different C−H rocking (below 1000 cm^−1^) and C−C stretching vibrations (above 1000 cm^−1^), which cannot be seen in ABS, HIPS, or HDPE. The 1455 cm^−1^ peak (wagging vibration of the −CH_3_ group) is in the vicinity of the 1451 cm^−1^ scissoring mode of −CH_2_ present in HIPS and ABS, thus not unique, while the 1375 + 1360 cm^−1^ peak associated with the −CH_3_ in-plane umbrella mode was very strong and unique for PP.

HDPE, on the other hand, has a rather simple spectrum, exhibiting peaks of C−H asymmetric and symmetric stretching vibrations 2914 and 2846 cm^−1^, respectively, in the MWIR, with the latter one being exclusive to PE, yet in the vicinity of the 2922 cm^−1^ (ABS), 2920 cm^−1^ (HIPS) and 2916 cm^−1^ (PP) so its behavior in a polymer mixture is not easily predictable. In the LWIR (Table S6), the split −CH− bending peaks located at 1471 + 1461 cm^−1^ are characteristic of PE [45], but they are in the vicinity of other polymer peaks (e.g., 1492 and 1451 cm^−1^ for ABS and HIPS, 1455 cm^−1^ for PP), thus not easily recognizable in a mixture. The same holds true for the split −CH_2_ rocking peaks located at 731 + 718 cm^−1^, which are also intense and specific to HDPE [45] but not visible in the herein mixture. Also, the ones detected at 1367, 1350, and 1304 cm^−1^, which are already “hard-to-see” peaks in the virgin HDPE, were not discernible. Ultimately, regardless of the homogenization strategy, HDPE was not visible mainly due to its low quantity (1% wt.) and the overlapping of peaks.

#### 3.3.4. Evaluate the Efficiency to Define Melt Processability

MFR of recyclates can be significantly affected by thermal and mechanical degradation, chain scission (which increases MFR), and contamination during previous processing cycles, which can result in reduced mechanical properties and processability concerns. Accordingly, the model waste mixture consists of different polymers (ABS, HIPS, PP, and HDPE), and their inherent MFR differences impact the blend’s overall processability. By measuring MFR after homogenization (e.g., extrusion, injection molding), it is possible to evaluate the uniformity degree achieved in the melt flow behavior of the waste, which directly influences the feasibility of reprocessing and recyclate quality.

The MFR values (sample size ca. 25 g) determined for the model waste samples are included in Table 6. In contrast to the PP-RE sorted waste (Section 3.1), where only one MFR replicate out of five was acceptable based on the ISO 1133, almost all model waste samples were within the guideline, with only the extruded ones at 210 °C (MW-ABS500-EX210) and its combination with injection (MW-ABS500-EX210-IJ) possessing replicates outside of the Max–Min < 15%∙Average rule (in a red font in Table 6). When comparing the different homogenization techniques, it can be seen that the ground material (MW-ABS500-GR) presented a very high MFR (67.2 ± 3.0 g/10 min, RSD = 4.5%) compared to the rest of the model waste samples and interestingly when these samples were subsequently injection molded (MW-ABS500-GR-IJ) the relevant values decreased to 53.1 ± 2.5 g/10 min (RSD = 4.7%) and were in the vicinity of the MFR values of the other homogenization routes. The presence of lubricants (both internal, e.g., metal stearates, waxes, and external, e.g., fatty acids, silicones) that may leach out during grinding/milling can significantly increase the MFR, since friction is reduced between polymer chains or between polymer-processing equipment, leading to plasticization and therefore, a reduction in viscosity. This finding aligns with the DSC findings (Section 3.3.3) and the lower *T*_g_ for the ground materials compared to other model waste samples. When applying only extrusion (MW-ABS500-EX210, MW-ABS50-EX200), lower MFR distribution among the individual measurements was observed (RSDs = 1.9 and 3.9%, respectively) compared to grinding; combining extrusion with grinding (MW-ABS500-EX210-GR, MW-ABS500-EX200-GR) slightly decreased the MFR values to 50.0 and 49.3 g/10 min, respectively with similar RSD values; however, when combining it with injection molding (MW-ABS500-EX210-IJ, MW-ABS500-EX200-IJ), higher MFR values were obtained (55.7 and 54.5 g/10 min), indicating that further melt processing of the material can lead possibly to chain scission. The distribution of values was also increased for the injection molded samples, since RSDs increased from a 1.9 to 4.5% range to a higher value (8.5%) for MW-ABS500-EX210-IJ.

Thermal stability is also a crucial characteristic influencing melt processability, recyclability, and overall performance of polymeric materials. Especially for recycled plastics, thermal stability is often compromised due to oxidation, hydrolysis, or residual contaminants that can act as degradation catalysts. In the context of model waste, TGA (sample size ca. 20–30 mg) allows for a clear understanding of how a waste material withstands thermal processing and long-term application without significant degradation. All model waste samples presented a single degradation step with reproducible curves and no significant changes between the homogenization techniques could be observed (Table S7). All samples presented good thermal stability with *T*_d,5%,_ and *T*_d_ in the ranges of 369–383 °C and 429–434 °C, with reproducible results (RSDs = 0.3–1.1 and 0.2–0.4%, respectively). In terms of residues (W), it is noticeable that the ground samples (MW-ABS500-GR, MW-ABS500-EX210-GR, and MW-ABS500-EX200-GR) presented a value at approximately 2% wt., higher than the rest of the samples. Moreover, these ground samples (GR and EX-GR) presented as expected slightly lower *T*_d,5%,_ and *T*_d_ compared to injected dogbones (GR-IJ and EX-IJ) and pellets (EX) because ground material in the form of powder presents a larger contact area exposed to the temperature.

### 3.4. Establishing Proper Homogenization Procedures for Each Analytical Technique—Proof of Concept for the PP-Rich Waste

Based on the developed model waste methodology herein, best practices (Table 7) regarding sample preparation and specific homogenization procedure per analytical technique can be proposed, depending on the desired sensitivity on volatile compounds/additives detection, degree of homogeneity, limitations of the respective analytical methods, etc.

In this way, the sample preparation protocols proposed can serve as a frame for analyzing a real sorted PWS, as well as their respective recyclates after the selected recycling/upcycling strategy. In order to demonstrate this claim, PP-RE, characterized as received in Section 3.1, was homogenized via twin-screw extrusion (180/195/200/200/205/205/200/205 °C, 130 rpm) and then used for injection molding. The samples were reanalyzed in terms of MFR, tensile testing, and X-ray micro-computed tomography so as to juxtapose the results with their inhomogeneous counterpart.

MFR values present a narrow range (5.7–5.9 g/10 min), which was also manifested in low RSDs < 4% and SD = 0.2 g/10 min among the five replicates (Table 8). In contrast to its non-homogenized counterpart (Section 3.1, Table 3), the homogenized PP-rich waste stream was within the acceptable margins of the ISO 1133 standard, since the Max–Min from each data set did not surpass the 15% of its average, for all replicates. Consequently, the homogenization strategies presented herein rendered the MFR determination feasible and reliable.

**Figure 12:**
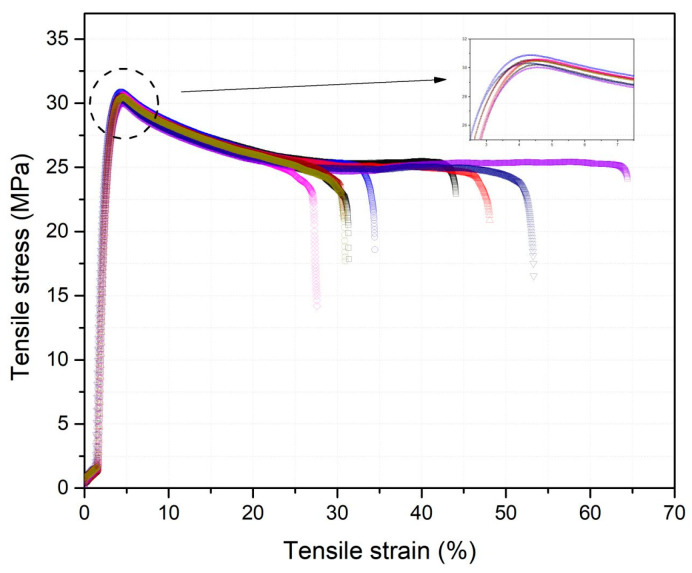
Tensile stress-strain curves of homogenized by melt extrusion and injection molding PP-rich EEE waste stream.

The homogeneity of PP-rich samples after extrusion and injection molding was visually apparent in comparison to sorted samples that showed multiple color variations, suggesting that there are not so distinct phases or regions with different compositions. The stress-strain curves of homogenized PP-RE material in Figure 12 revealed a significant decrease in the variability of tensile properties, especially the yield strength. As was mentioned earlier, the presence of distinct phases within the specimen, as well as discontinuities, acts as a stress concentrator and leads to premature failure and reduced mechanical performance. As a result, the homogenization of the dominant polymer PP with other polymeric contaminations leads to a continuous matrix that has a more uniform mechanical response and failure pattern.

Table 9 presents the overall tensile properties for ten 1BA specimens for the homogenized by extrusion and injection molding PP-RE. The significant increase in repeatability is evident by the decrease in RSD values. More specifically, *σ*_y_ presented low RSD values, equal to 0.9% (while for the respective unhomogenized one was 4.1%), for *ε*_y_ was 0.03% vs. 3.5% for the unhomogenized one, and finally *E* RSD values were 1.7% vs. 7.4%, respectively.

**Figure 13:**
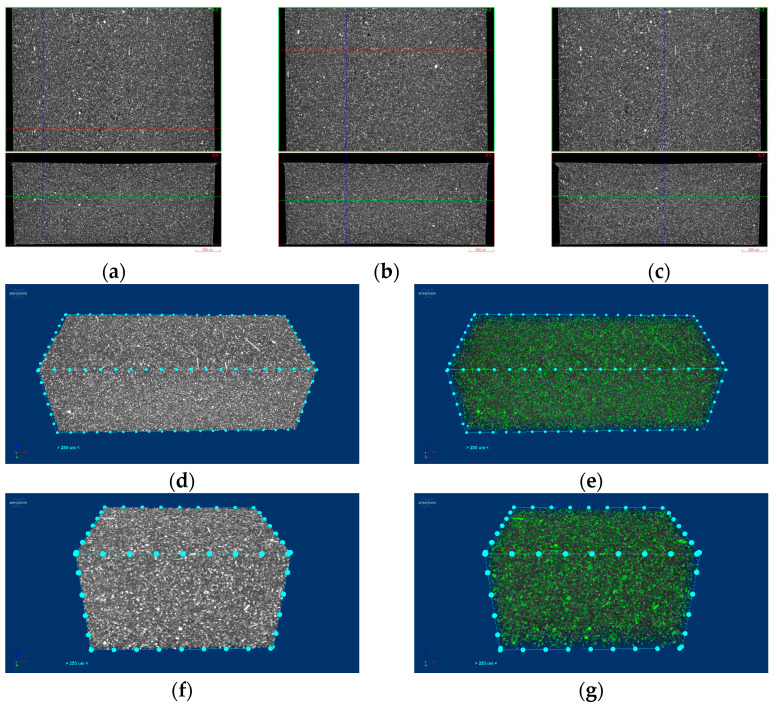
(**a**–**c**) Representative XZ and XY cross sections of the reconstructed grayscale slices of PP-rich homogenized samples (DataViewer software v.1.2.5.7, Bruker microCT); scale bar: 600 μm; (**d**,**f**) 3D visualization of PP-rich homogenized sample volume (CTvox, software v3.3.1, Bruker microCT); (**e**,**g**) Segmentation of dispersed particles (highlighted in green).

The effect of homogenization is also evident when analyzing the homogenized samples via micro-CT, where the dispersed phase of particles within a 6–20 μm diameter range is evenly distributed throughout the samples’ volume, while indications of insufficient blending among different colored flakes have been eliminated, with the polymer matrix presenting consistent X-ray attenuation levels (Figure 13a–c). High X-ray absorption particles of large sizes (100–200 μm) previously observed in samples originating from sorted PP-rich EEE waste streams have also been removed or fragmented, as observed in Figure 13d–g.

## 4. Conclusions

This work describes the potential, as well as the limitations, of the existing sampling and sample preparation protocols, analytical techniques, and common practices applied in the plastic recycling industry. These limitations can be mitigated by combining a sampling plan, following the established standards, along with our recommendations tailored to specific analytical objectives. To realize this, a mixture of polymers (ABS, HIPS, PP, and HDPE) of known composition was used, simulating typical sorted ABS-rich electrical and electronic equipment waste streams, containing also a representative brominated flame retardant. Different homogenization/blending techniques were examined to develop an optimal sampling and sample preparation scheme per analytical technique. The objectives involved establishing a correlation of the sample preparation steps with low deviation values between replicates, as well as determining the proximity of the targeted additives/substances content to the actual values. Importantly, our findings showed that the suitability of a sample preparation pathway strongly depends on the analytical technique. Cryogenic grinding was found to be most appropriate for the reliable detection and quantification of non-polymeric contaminants like BFRs via HPLC-MS, as it minimized degradation of volatile or thermally sensitive species. In contrast, melt-based homogenization via extrusion and injection molding significantly improved reproducibility in terms of melt flow behavior and processability (MFR), thermal (DSC), and mechanical performance by reducing material heterogeneity, incompatibility between polymers, etc., albeit at the risk of partial additive degradation. Combined preparation routes, such as extrusion followed by grinding, offered a balanced solution for elemental (XRF) and bulk analyses (ATR-FTIR, TGA, GPC) where both homogeneity and medium representativeness were required. The herein proposed homogenization strategies can be crucial for recycling purposes in terms of assessing contamination levels and waste degradation extent, choosing suitable processing techniques and their temperature window, and eventually evaluating and selecting the appropriate recycling/upcycling strategy. This will not only increase the precision and reliability of the obtained analytical results but also potentially lead to more efficient recycling technologies and policies, paving the way towards a more sustainable plastics future.

## Figures and Tables

**Table 1:** Nomenclature of model waste samples prepared with different blending/homogenization techniques containing a brominated flame retardant (tetrabromobisphenol A, TBBPA).

Sample Preparation Technique	Sample	TBBPA (ppm)	*T*_melt_ (°C)
**Masterbatch—Extrusion**	10% wt. BFR-ABS	100,000	—
**Cryogenic Grinding (GR)**	MW-ABS50(0)-GR	50(0)	—
**Cryogenic Grinding (GR) + Injection (IJ)**	MW-ABS500-GR-IJ	500	—
**Extrusion (EX)**	MW-ABS50(0)-EX210	50(0)	210
	MW-ABS500-EX200	500	200
**Extrusion (EX) + Cryogenic Grinding (GR)**	MW-ABS50(0)-EX210-GR	50(0)	210
	MW-ABS500-EX200-GR	500	200
**Extrusion (EX) + Injection (IJ)**	MW-ABS500-EX210-IJ	500	210
	MW-ABS500-EX200-IJ	500	200

*T*_melt_: melt temperature measured.

**Table 2:** Elemental analysis of sorted PP-rich EEE waste stream from refrigerators (PP-RE) determined via XRF spectroscopy.

Element	P	Si	Ca	Ti	Br	Sb	Fe	Zn	Pb	Cd	Cr
**Concentration (ppm)**	92	40,814	33,656	8478	416	225	806	197	16	<5	16
**SD (ppm)**	11	6561	7879	852	54	48	54	27	5	2	11
**RSD (%)**	11.9	16.1	23.4	10.0	12.9	21.3	6.7	13.7	31.3	40	68.8

**Table 3:** MFR values of five replicates of sorted PP-rich EEE waste stream from refrigerators (PP-RE).

Sample (x_i_)	MFR (230 °C, 2.16 kg) g/10 min				
	Replicate 1	Replicate 2	Replicate 3	Replicate 4	Replicate 5
x_1_	7.7	9.1	8.7	9.1	9.9
x_2_	8.8	7.2	8.1	10.5	9.4
x_3_	8.3	8.0	8.7	9.4	8.7
x_4_	8.7	7.9	7.5	9.6	9.3
x_5_	7.8	8.8	8.4	10.0	9.7
x_6_	9.3	7.6	7.9	9.9	8.6
x_7_	7.9	5.9	7.8	9.2	10.1
x_8_	7.7	6.8	8.3	9.9	9.6
x_9_	7.6	6.5	8.0	11.0	10.4
**Average (g/10 min)**	8.2	7.5	8.2	9.8	9.5
**SD (g/10 min)**	0.6	1.1	0.4	0.6	0.6
**RSD (%)**	7.3	14.0	5.0	6.3	6.2
**Max−Min (g/10 min)**	**1.7**	**3.2**	1.2	**1.9**	**1.7**
**15% Average (g/10 min)**	1.2	1.1	1.2	1.5	1.4

**Table 4:** Tensile testing values of ten replicates of sorted PP-rich EEE waste stream from refrigerators (PP-RE).

Specimen (x_i_)	*E* (MPa)	*σ*_y_ (MPa)	*ε*_y_ (%)
x_1_	1501	26.5	6.1
x_2_	1340	24.6	5.7
x_3_	1524	26.3	5.9
x_4_	1524	27.0	6.1
x_5_	1629	27.6	6.2
x_6_	1589	27.4	6.4
x_7_	1716	28.2	6.0
x_8_	1674	28.4	6.1
x_9_	1411	26.6	5.9
x_10_	1576	26.6	5.9
**Average**	1548	26.9	6.0
**SD**	115	1.1	0.2
**RSD (%)**	7.4	4.1	3.5

**Table 5:** GPC results of ABS (predominant polymer) in the model waste samples prepared via different blending/homogenization techniques.

Sample	${\bar{M}}_{n}$ (g/mol)	${\bar{M}}_{w}$ (g/mol)	Đ
ABS (virgin polymer)	25,800	123,000	4.8
MW-ABS500-GR	24,700	118,000	4.8
MW-ABS500-GR-IJ	22,600	123,000	5.4
MW-ABS500-EX210	24,200	126,000	5.2
MW-ABS500-EX200	25,100	126,000	5.0
MW-ABS500-EX210-GR	24,000	126,000	5.3
MW-ABS500-EX200-GR	23,700	126,000	5.3
MW-ABS500-EX210-IJ	24,300	122,000	5.0

**Table 6:** MFR results of the model waste samples prepared via different blending/homogenization techniques.

Sample	MFR (220 °C, 10 kg) g/10 min				
	Average	SD	RSD (%)	Max−Min	15%∙Average ^a^
MW-ABS500-GR	67.2	3.0	4.5	5 replicates < 15%∙Average	4.2–9.4
MW-ABS500-GR-IJ	53.1	2.5	4.7	5 replicates < 15%∙Average	1.0–4.2
MW-ABS500-EX210	51.9	2.0	3.9	**3 replicates < 15%∙Average**	3.3–47.7
MW-ABS500-EX200	53.7	1.0	1.9	5 replicates < 15%∙Average	1.7–7.2
MW-ABS500-EX210-GR	50.0	1.3	2.5	5 replicates < 15%∙Average	1.4–3.7
MW-ABS500-EX200-GR	49.3	1.0	1.9	5 replicates < 15%∙Average	1.2–2.2
MW-ABS500-EX210-IJ	55.7	4.7	8.5	**4 replicates < 15%∙Average**	1.8–21.6
MW-ABS500-EX200-IJ	54.5	2.2	4.0	5 replicates < 15%∙Average	0.9–3.6

^a^ For the 5 replicates.

**Table 7:** Proposed sample preparation strategy per analytical technique.

Sample Preparation Technique	Analytical Technique	Waste Characteristic Analyzed
Cryogenic grinding	HPLC-MS	Quantified non-polymeric contaminants
Extrusion + Cryogenic grinding	XRF	Elemental composition
Extrusion + Injection	MFR DSC Tensile testing	Melt flow behavior and processability Melting and crystallization Mechanical performance
All	ATR-FTIR TGA GPC	Detected polymeric contaminants Thermal stability Degradation extent via molecular weight determination

**Table 8:** MFR results of homogenized by extrusion and injection molding PP-rich EEE waste stream from refrigerators (PP-RE).

Sample (x_i_)	MFR (230 °C, 2.16 kg) g/10 min				
	Replicate 1	Replicate 2	Replicate 3	Replicate 4	Replicate 5
x_1_	6.1	6.2	6.0	6.0	5.9
x_2_	6.0	6.1	6.0	6.1	5.9
x_3_	6.0	6.1	6.0	6.0	5.9
x_4_	6.1	6.0	6.0	6.0	5.9
x_5_	5.9	5.9	6.1	6.0	5.8
x_6_	5.9	5.9	5.7	6.0	5.6
x_7_	6.0	6.0	5.6	6.0	5.7
x_8_	5.9	5.9	5.6	6.1	5.6
x_9_	5.8	5.8	5.5	5.8	5.6
**Average (g/10 min)**	5.8	5.9	5.7	5.9	5.7
**SD (g/10 min)**	0.2	0.2	0.2	0.2	0.2
**RSD (%)**	4.2	3.4	4.3	3.1	3.1
**Max−Min (g/10 min)**	**0.8**	**0.6**	**0.6**	**0.6**	**0.5**
**15% Average (g/10 min)**	0.9	0.9	0.9	0.9	0.9

**Table 9:** Tensile testing values of ten replicates of homogenized by extrusion and injection molding PP-rich EEE waste stream from refrigerators (PP-RE).

Specimen (x_i_)	*σ*_y_ (MPa)	*ε*_y_ (%)	E (MPa)
x_1_	30.2	4.5	2270
x_2_	30.0	4.2	2265
x_3_	30.6	4.4	2295
x_4_	30.9	4.2	2270
x_5_	30.6	4.5	2249
x_6_	30.6	4.6	2209
x_7_	30.0	4.5	2161
x_8_	30.3	4.2	2265
x_9_	30.5	4.4	2253
x_10_	30.4	4.4	2249
**Average**	30.4	4.4	2249
**SD**	0.3	0.2	38
**RSD (%)**	0.9%	0.0%	1.7%

## Data Availability

The data presented in this study are available on request from the corresponding authors due to data protection and confidentiality requirements within a European Union–funded project.

## Supplementary Materials

The following supporting information can be downloaded at: https://www.mdpi.com/article/10.3390/polym18030409/s1, Figure S1: Indicative pictures of (a) the real sorted PP-rich EEE waste stream from refrigerators (PP-RE) in the form of flakes containing different polymers of different colors, (b) injection-molded specimens produced directly from the real sorted PP-rich EEE waste stream from refrigerators (PP-RE); Figure S2: ATR-FTIR spectra of five different colored flakes of sorted PP-rich EEE waste stream from refrigerators (PP-RE) in the (a) 2800–4000 cm^−1^ and (b) 400–2000 cm^−1^ regions; Figure S3: DSC thermograms of five different colored flakes of sorted PP-rich EEE waste stream from refrigerators (PP-RE) during (a) cooling and (b) 2nd heating cycle; Table S1: Composition calculated for 1 kg of model waste containing 500 ppm TBBPA. Comparison of calculated weight fractions (wt. %), SD, and RSD values of each component in the sorted waste stream (20,000 flakes, 0.05 g/flake) and in the ground sorted waste stream (400,000 particles, size ca. 750 μm); Table S2: Signals in cm^−1^ of TBBPA, 10% wt. BFR-ABS, and model waste samples in the MWIR; Table S3: Signals in cm^−1^ of TBBPA, 10% wt. BFR-ABS, and model waste samples in the LWIR; Table S4: DSC results of the model waste samples prepared via different blending/homogenization techniques; Table S5: Signals in cm^−1^ of virgin polymers and model waste samples in the MWIR; Table S6: Signals in cm^−1^ of virgin polymers and model waste samples in the LWIR; Table S7: TGA results of the model waste samples prepared via different blending/homogenization techniques.
