# Supplementary material for: Taming Waste Heterogeneity for Plastics Circularity with Optimized Sample Preparation Protocols for Quality Assessment
Source: Polymers (Basel). 2026 Feb 4;18(3):409. doi: 10.3390/polym18030409 (PMC12899086; doi:10.3390/polym18030409)
Supplement: Supplementary file 1 [file polymers-18-00409-s001.zip › polymers-4038813-supplementary.pdf]

Supplementary information for

Taming Waste Heterogeneity for Plastics Circularity with Optimized Sample Preparation Protocols for Quality Assessment

Christos Panagiotopoulos [1], Christina Podara [2], Eleni Gkartzou [2], Melpo Karamitrou [2], Tatjana Kosanovic-Milickovic [2], Mara Silber [3], Lars Meyer [3], Bernhard von Vacano [3], Ana Rita Carvalho Neiva [4], Jan-Hendrik Knoop [5], Asunción Martínez-García [6], Ana Ibáñez-García [6], Silvia Pavlidou [7], Leila Poudeh [8], Costas A. Charitidis [2,*] and Stamatina N. Vouyiouka [1,*]

[1] Laboratory of Polymer Technology, School of Chemical Engineering, Zographou Campus, National Technical University of Athens, 157 72 Athens, Greece; chpanagiotopoulos@mail.ntua.gr

[2] Research Lab of Advanced, Composites, Nanomaterials and Nanotechnology, School of Chemical Engineering, Zographou Campus, National Technical University of Athens, 157 72 Athens, Greece; cpodara@chemeng.ntua.gr (C.P.); egartzou@chemeng.ntua.gr (E.G.); mkaramitru@chemeng.ntua.gr (M.K.); tkosanovic@chemeng.ntua.gr (T.K.-M.)

[3] Group Research, BASF SE, 67056 Ludwigshafen am Rhein, Germany; mara.silber@basf.com (M.S.); lars.meyer@basf.com (L.M.); bernhard.von-vacano@basf.com (B.v.V.)

[4] Coolrec Plastics BV, Van Hilststraat 7, 5145 RK Waalwijk, The Netherlands; Ana.Rita.Neiva@coolrec.com

[5] Fraunhofer Institute for Process Engineering and Packaging IVV, 85354 Freising, Germany; jan-hendrik.knoop@ivv.fraunhofer.de

[6] Innovative Materials and Manufacturing Area, AIJU, Technological Institute for Children's Products and Leisure, 03440 Ibi, Alicante, Spain; sunymartinez@aiju.es (A.M.-G.); anaibanyez@aiju.es (A.I.-G.)

[7] MIRTEC S.A., 76th km Athens-Lamia National Road, 320 09 Schimatari, Greece; s.pavlidou@mirtec.gr

[8] Central R&D, Polymer & Chemistry Department, Beko Corporate (Arçelik Global), 34950 Tuzla, Istanbul, Turkey; leila.poudeh@beko.com

* Correspondence: charitidis@chemeng.ntua.gr (C.A.C.); mvuyiuka@central.ntua.gr (S.N.V.); Tel.: +30-210-772-4046 (C.A.C.); +30-210-772-2249 (S.N.V.)

a)

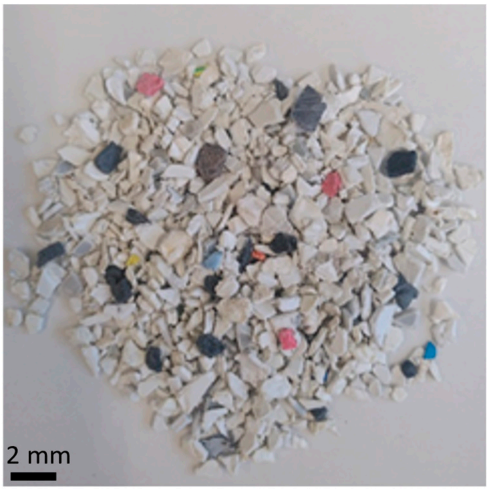

b)

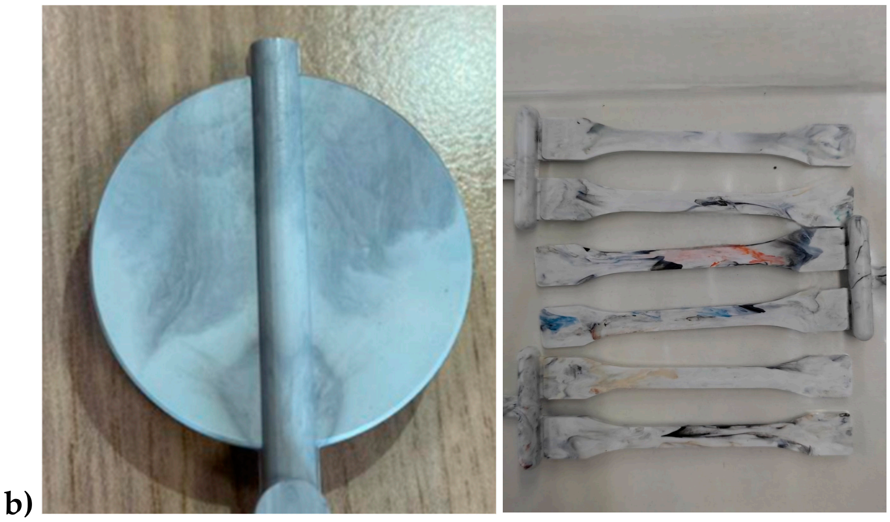

Figure S1. Indicative pictures of **a)** the real sorted PP-rich EEE waste stream from refrigerators (PP-RE) in the form of flakes (2–4 mm range of size, thickness 1–2 mm) containing different polymers of different colors, and **b)** injection-moulded specimens produced directly from the real sorted PP-rich EEE waste stream from refrigerators (PP-RE).

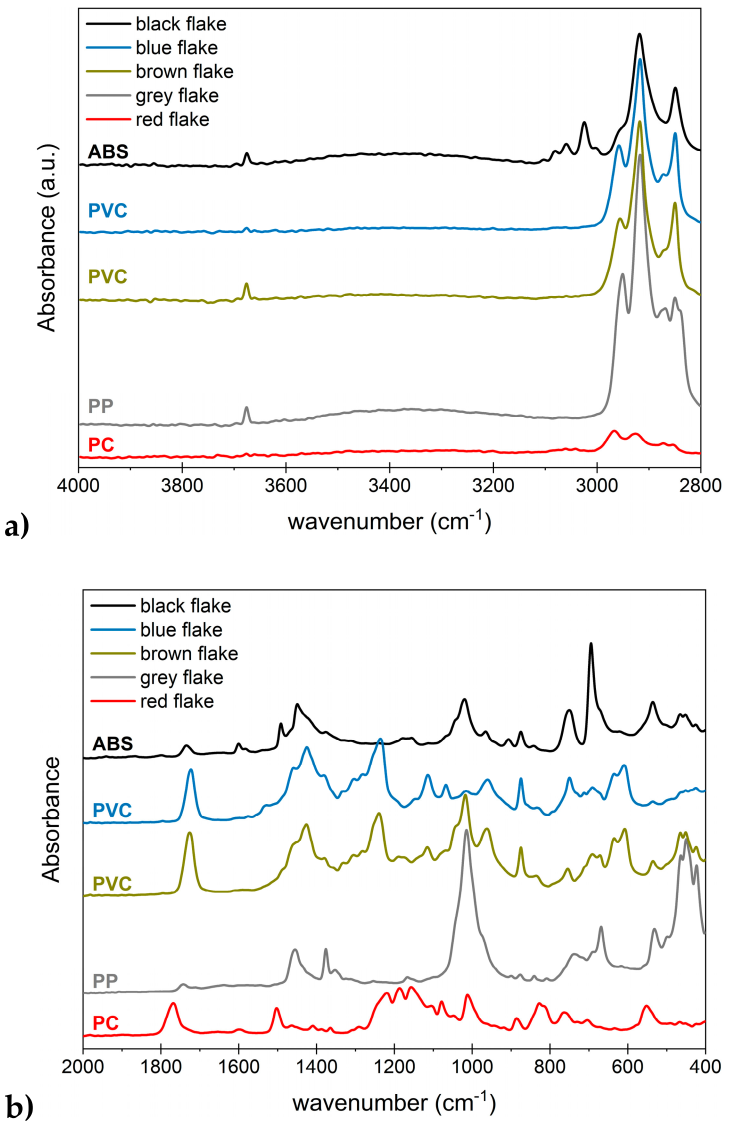

Figure S2. ATR-FTIR spectra of five different colored flakes of sorted PP-rich EEE waste stream from refrigerators (PP-RE) in the a) 2800–4000 cm^{-1} and b) 400–2000 cm^{-1} regions.

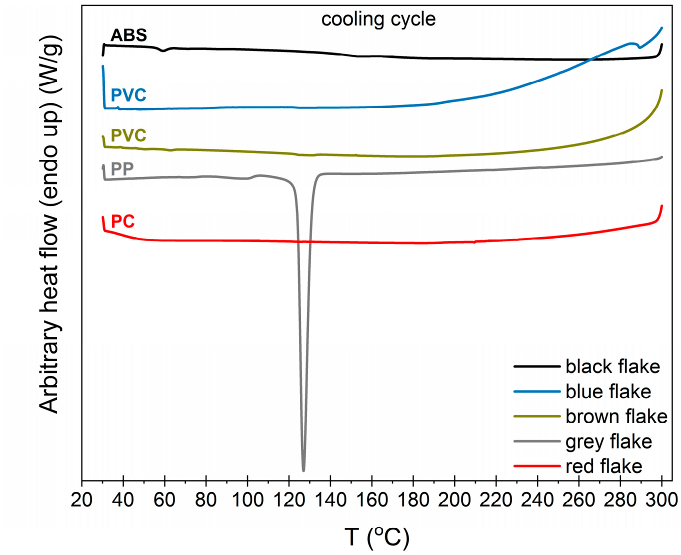

a)

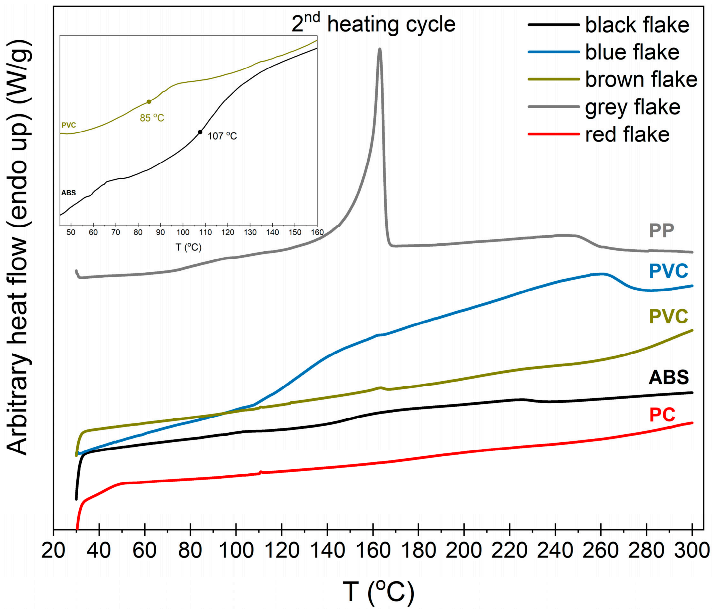

b)

Figure S3. DSC thermograms of five different colored flakes of sorted PP-rich EEE waste stream from refrigerators (PP-RE) during a) cooling and b) 2nd heating cycle.

Table S1. Composition calculated for 1 kg of model waste containing 500 ppm TBBPA. Comparison of calculated weight fractions (wt. %), SD, and RSD values of each component in the sorted waste stream (20000 flakes, 0.05 g/flake) and in the ground sorted waste stream (400000 particles, size ca. 750 μm).

		**Sorted waste stream (20000 flakes)**			**Ground waste stream (400000 particles)**		
	**Nom. wt. %**	**Calc. wt. %**	**SD %**	**RSD %**	**Calc. wt. %**	**SD %**	**RSD %**
ABS	90.5	90.3	3.9	4	90.4	0.13	0.14
HIPS	6.0	7.0	2.7	39	6.1	0.10	1.6
PP	2.0	1.8	1.7	94	2.0	0.06	3.0
HDPE	1.0	0.5	0.8	160	1.0	0.06	6.0
TBBPA	0.5	0.3	0.7	233	0.5	0.03	6.0

Nom. wt. %: Nominal weight fraction; Calc. wt. %: Calculated weight fraction.

Table S2. Signals in cm^{-1} of TBBPA, 10 % wt. BFR-ABS, and model waste samples in the MWIR.

Sample	TBBPA (ppm)	3520	3480	3070	2990	2970	2950
TBBPA*		3515	3475	3074	2989	2972	2947
10 % wt. BFR-ABS	10000						
MW-ABS500-GR	500						2951
MW-ABS500-GR-IJ	500						2949
MW-ABS500-EX210	500						2950
MW-ABS500-EX200	500						2950
MW-ABS500-EX210-GR	500						2951
MW-ABS500-EX200-GR	500						2951
MW-ABS500-EX210-IJ	500						2951
MW-ABS500-EX200-IJ	500						2951

*TBBPA peaks from literature [1]; Blue: outstandingly intense; red: strong; orange: moderate; yellow: weak; pale yellow: very weak, hard-to-see

Table S3. Signals in cm^{-1} of TBBPA, 10 % wt. BFR-ABS, and model waste samples in the LWIR.

**Sample**	**TBBPA (ppm)**	**1560**	**1470**	**1400**	**1320**	**1280**	**1250**	**1230**	**1200**	**1170**	**1160**	**1130**	**940**	**890**	**870**	**780**	**730**	**710**	**650**	**620**
TBBPA*		1555	1472	1396	1312	1272	1238	1226	1197	1173	1157	1132	940	887	867	780	731	706	648	616
10 % wt. BFR-ABS	10000	1555	1472	1393	1317	1278	1245				1159	1130			870		733			

*TBBPA peaks from literature [1]; Blue: outstandingly intense; red: strong; orange: moderate; yellow: weak; pale yellow: very weak, hard-to-see

Table S4. DSC results of the model waste samples prepared *via* different blending/homogenization techniques.

	T_g (°C)			Endothermic peak 1 (°C)			Endothermic peak 2 (°C)		
**Sample**	**Average**	**SD**	**RSD (%)**	**Average**	**SD**	**RSD (%)**	**Average**	**SD**	**RSD (%)**
MW-ABS500-GR	94.8	3.1	3.3	131.2	0.3	0.2	162.8	0.5	0.3
MW-ABS500-GR-IJ	105.5	0.9	0.9	130.8	0.4	0.3	165.4	0.4	0.2
MW-ABS500-EX210	105.4	0.4	0.4	128.2	0.2	0.1	165.1	0.1	0.1
MW-ABS500-EX200	107.0	1.1	1.0	129.8	1.1	0.9	166.1	0.5	0.3
MW-ABS500-EX210-GR	105.4	0.4	0.4	128.2	0.2	0.2	165.1	0.1	0.1
MW-ABS500-EX200-GR	103.0	0.4	0.4	127.8	0.1	0.1	165.5	0.3	0.2
MW-ABS500-EX210-IJ	105.8	0.8	0.8	130.6	0.1	0.1	165.3	0.3	0.2
MW-ABS500-EX200-IJ	106.4	0.4	0.4	130.4	0.4	0.3	165.2	0.2	0.1

Table S5. Signals in cm^{-1} of virgin polymers and model waste samples in the MWIR.

Sample	3080	3060	3030	3000	2950	2920	2910	2870	2850	2840	2720	2240
ABS	3083	3060	3027	3004		2922			2852			2237
HIPS	3080	3060	3025	3000		2920			2850			
PP					2949	2916		2867		2840	2723	
HDPE							2914		2846			
MW-ABS500-GR	3083	3061	3027	3004	2951	2920			2850			2237
MW-ABS500-GR-IJ	3083	3062	3027	3004	2949	2920			2850			2237
MW-ABS500-EX210	3083	3062	3027	3004	2950	2920			2850			2239
MW-ABS500-EX200	3083	3062	3027	3002	2950	2920			2850			2237
MW-ABS500-EX210-GR	3083	3062	3027	3004	2951	2920			2850			2237
MW-ABS500-EX200-GR	3083	3062	3027	3004	2951	2920			2850			2237
MW-ABS500-EX210-IJ	3083	3060	3027	3004	2951	2920			2850			2237
MW-ABS500-EX200-IJ	3082	3062	3027	3004	2951	2918			2850			2237

Blue: outstandingly intense; red: strong; orange: moderate; yellow: weak; pale yellow: very weak, hard-to-see

Table S6. Signals in cm^{-1} of virgin polymers and model waste samples in the LWIR.

Sample	1600	1490	1470	1460	1450	1380	1370	1360	1350	1300	1170	1100	1030	1000	970	940	910	900	840	810	760	750	730	720	700	690
ABS	1601	1494			1451								1029		965		910		845		757				698	
HIPS	1601	1492			1451								1029		965		908		842			749				693
PP				1455		1375		1360		1303	1167	1103		998	973	940		899	840	809						
HDPE			1471	1461			1367		1350	1304													731	718		
MW-ABS500-GR	1601	1494			1453	1375		1362					1029		965		912		844		759				698	
MW-ABS500-GR-IJ	1601	1494			1453	1375		1362					1029		965		910		844		759				698	
MW-ABS500-EX210	1601	1494			1453	1375		1362					1029		965		910		844		759				698	
MW-ABS500-EX200	1601	1494			1453	1375		1360					1029		965		910		844		759				698	
MW-ABS500-EX210-GR	1601	1494			1453	1375		1360					1029		965		912		844		759				698	
MW-ABS500-EX200-GR	1601	1494			1453	1375		1362					1029		965		910		844		759				698	
MW-ABS500-EX210-IJ	1601	1494			1453	1375		1362					1029		965		912		844		759				698	
MW-ABS500-EX200-IJ	1601	1494			1453	1375		1360					1029		965		912		846		759				698	

Blue: outstandingly intense; red: strong; orange: moderate; yellow: weak; pale yellow: very weak, hard-to-see

Table S7. TGA results of the model waste samples prepared *via* different blending/homogenization techniques.

	$T_{d,5\%}$ (°C)			T_d (°C)			Residue (W, %) (@ 300 °C)		
**Sample**	**Average**	**SD**	**RSD (%)**	**Average**	**SD**	**RSD (%)**	**Average**	**SD**	**RSD (%)**
MW-ABS500-GR	366.5	5.0	1.4	431.4	1.3	0.3	2.0	0.2	10.8
MW-ABS500-GR-IJ	382.5	3.0	0.8	433.9	1.5	0.4	1.4	0.2	12.4
MW-ABS500-EX210	368.9	4.1	1.1	429.1	1.8	0.4	1.5	0.1	7.9
MW-ABS500-EX200	374.7	2.9	0.8	431.4	1.7	0.4	1.7	0.0	2.3
MW-ABS500-EX210-GR	381.4	1.3	0.4	433.5	1.3	0.3	2.0	0.2	10.8
MW-ABS500-EX200-GR	378.7	1.1	0.3	432.3	1.4	0.3	1.9	0.2	12.1
MW-ABS500-EX210-IJ	381.2	1.2	0.3	432.7	1.2	0.3	1.4	0.0	1.4
MW-ABS500-EX200-IJ	380.9	2.4	0.6	430.5	1.1	0.2	1.5	0.2	11.9

References

1. Signoret, C.; Caro-Bretelle, A.-S.; Lopez-Cuesta, J.-M.; Ienny, P.; Perrin, D. Alterations of Plastics Spectra in MIR and the Potential Impacts on Identification towards Recycling. *Resources, Conservation and Recycling* **2020**, *161*, 104980, doi:10.1016/j.resconrec.2020.104980.
